# Supplementary material for: MicroRNAs mediate precise control of spinal interneuron populations to exert delicate sensory-to-motor outputs
Source: eLife. 2021 Mar 31;10:e63768. doi: 10.7554/eLife.63768 (PMC8075582; doi:10.7554/eLife.63768)
Supplement: Supplementary file 1. [file elife-63768-supp1.docx]

**Supplementary tables file**

| **Supplementary file 1a. sgRNA sequences used in this work** | | |
| --- | --- | --- |
| **Target** | **Sequence (5’~3’)** |  |
| *Mir449* sgRNA1 | TAATACGACTCACTATAGGG (T7 promoter) + GGG (Transcription starting cue) + **CTGGGAAGCGCCCGCTAAGC (target deletion)** + GTTTTAGAGCTAGAAATAGC (tracrRNA) | |
| *Mir449* sgRNA2 | TAATACGACTCACTATAGGG (T7 promoter) + GGG (Transcription starting cue) + **CATAATAGTACCCCTCTTCA (target deletion)** + GTTTTAGAGCTAGAAATAGC (tracrRNA) | |

| **Supplementary file 1b. List of primers for mouse genotyping** | | |
| --- | --- | --- |
| **Gene name** | **Forward Primer** | **Reverse Primer** |
| *Mir34a* WT | ATACCCTGGATCTCCAACAG | ACAAGACCCTCACCTGAATG |
| *Mir34a* KO | ATACCCTGGATCTCCAACAG | GCCATCCTGTTGAGGGACTA |
| *Mir34a* flox | ATACCCTGGATCTCCAACAG | ACAAGACCCTCACCTGAATG |
| *Mir34bc* WT | ATGACTTTACGGGGTTGACAG | AAATTCCTCCGACTGAGCCT |
| *Mir34bc* KO | ATGACTTTACGGGGTTGACAG | TTGCGGGAAGAAGGACTC |
| *Cre* | ATGGACATGTTCAGGGATC | CAGCCACCAGCTTGCATGA |
| *GFP* | CCCTGAAGTTCATCTGCACCAC | TTCTCGTTGGGGTCTTTGCTC |
| *Mir449* WT | GTTCCCCCAAGGTGAGTTTT | CCTGACACACCCACACTTCA |
| *Mir449* KO | GTTCCCCCAAGGTGAGTTTT | TGGAAACTTGGGCAAATACA |

| **Supplementary file 1c. List of primers for quantitative RT-PCR analyses** | | |
| --- | --- | --- |
| **Gene name** | **Forward Primer** | **Reverse Primer** |
| *Cdc20b* | AGCGAAATCAAGGTTCCCCC | GGAGGACCCTCTTCCACAGA |
| *Gapdh* | TGACCACAGTCCATGCCATC | GACGGACACATTGGGGGTAG |

| **Supplementary file 1d. List of primers for TOPO cloning** | | |
| --- | --- | --- |
| **Gene name** | **Forward Primer** | **Reverse Primer** |
| *Mir34a* | CACCCTTCAGCTTCCAGTTTTTACTTCAG | CAACACACATCATACAGTCTCCTTC |
| *Mir34bc* | CACCTTGCGGGAAGAAGGACTC | ATGACTTTACGGGGTTGACAG |
| *Mir449abc* | CACCCTATAGAAGGCATTTGCAGGAATTA | AATGCTTTGATAATGTCAAGCTAGG |

Forward primer contains CACC that is necessary for directional cloning

| **Supplementary file 1e. List of primers for the luciferase analyses** | | | |
| --- | --- | --- | --- |
| **Gene name** | | **Forward Primer** | **Reverse Primer** |
| *Satb2* 3'UTR WT | | AACG+CTCGAG+TGGGTCCAAGGACTTTCTGT | TTTA+GCGGCCGC+TCTGCACACCTACCAAAGCA |
| *Satb2* 3'UTR Mut1 | | ATAAgtgacggtAAAAGATGAGAGACAGGAGG | CTTTTaccgtcacTTATTTTGTTAAATACATACCTC |
| *Satb2* 3'UTR Mut2 | | ATTTgtgacggtAGTTTGCAGTGGTTCTAAG | GCAAACTaccgtcacAAATTAAAAAATAAAAGGC |
| *Satb2* 3'UTR Mut3 | | CACTAtcactatATTTTTAACTTATGTGCATTG | GTTAAAAATatagtgaTAGTGATAACAAAGATAGTTG |
| *Satb2* 3'UTR Mut4 | | GTGTaatcgatCAGAGAATGTAAATTAAAAAATGATCTTGG | CTCTGatcgattACACTTGCTGTTTCTT |
| *Satb1* 3'UTR WT | | AACGCTCGAGTCCAGGATAAAAACGCCAAC | TTTA+GCGGCCGCAACACACACCTTCCACCACA |
| *Satb1* 3'UTR Mut1 | | ATGGgtgacggGTAATTAAAATTTGTTTTTTTATTTTGG | TTACccgtcacCCATACAATACAAGGCATTTGTTG |
| *Satb1* 3'UTR Mut2 | | AATGacgactaTAGGAGTGGTTATTGACTGTAAG | CCTAtagtcgtCATTTCTGCATAAAGAAAACTGTG |
| *Satb1* 3'UTR Mut3 | | GAAAtcgttgaGAACACTCCCTGAGATACTC | GTTCtcaacgaTTTCAGAACCCACAAGCTATC |
| *Satb1* 3'UTR Mut4 | | CCCCatcactaCCACATTGTCCCCAGCTC | GTGGtagtgatGGGGAAATCTTCTGAAATACGC |
| *Satb1* 3'UTR Mut5 | | CAAGtcgttgaAAAATGTGTATGGCCTGTGC | TTTTtcaacgaCTTGCTGAAAATATCACCAGGC |
| Forward primer XhoI cutting site: CTCGAG | | | |
| Reverse primer NotI cutting site: GCGGCCGC | | | |
| Mutated targeted binding sites were presented as lowercase | | | |

| **Supplementary file 1f. List of controlled mice used in the representative figures and statistical analyses** | |
| --- | --- |
| **Figure** | **Genotypes of the controlled littermates** |
| *Fig. 2B and 2C* | All the combo litters crossing from the Fig. 2A strategy |
| *Fig. 3B and Figure 3—figure supplement 2B* | 34bc^+/-^;449^-/-^ & 34bc^-/-^ & 34a^-/-^;449^-/-^ & 34a^-/-^;34bc^+/-^;449^-/-^ & 34a^-/-^;34bc^-/-^ |
| *Fig. 3C and 3D* | 34a^-/-^;34bc^-/-^ & 34bc^-/-^ & 34a^-/-^;449^-/-^ & 34a^-/-^;34bc^+/-^;449^-/-^ & 34bc^+/-^;449^-/-^ |
| *Fig. 3E~H and Figure 3—figure supplement 2C~H* | 34bc^+/-^;449^-/-^ & 34bc^-/-^;449^+/-^ & 449^-/-^ & 34bc^+/-^;449^+/-^ & 449^+/-^ & 34a^-/-^;34bc^+/-^;449^-/-^ & 34a^-/-^;449^-/-^ |
| *Fig. 4B~D* | 34bc^-/-^ & 34a^-/-^;34bc^-/-^ & 449^+/-^ & 449^-/-^ & 34bc^+/-^;449^+/-^ & 34bc^+/-^;449^-/-^ & 34bc^-/-^;449^+/-^ & 34a^-/-^;449^-/-^ & 34a^-/-^;34bc^+/-^;449^-/-^ |
| *Fig. 5E* | 34bc^+/-^;449^-/-^ |
| *Fig. 5F* | 34a^-/-^;34bc^-/-^;449^+/-^ & 34a^-/-^;34bc^-/-^ & 34a^-/-^;449^-/-^ |
| *Fig. 5G~H* | 34a^-/-^;34bc^+/-^;449^-/-^& 34a^-/-^;449^-/-^ & 34bc^+/-^;449^-/-^ & 34bc^+/-^;449^+/-^ & 34bc^-/-^ & 449^-/-^ |
| *Fig. 6A* | 34bc^+/-^;449^-/-^ |
| *Fig. 6B~6D* | 34a^-/-^;34bc^+/-^;449^-/-^ & 34a^-/-^;449^-/-^ & 34bc^+/-^;449^-/-^ |
| *Fig. 6E and 6F* | 34a^-/-^;34bc^+/-^;449^-/-^& 34bc^+/-^;449^-/-^ & 34a^-/-^;449^-/-^ |
| *Figure 2—figure supplement 1A* | 34a^-/-^;34bc^-/-^;449^+/-^ |
| *Figure 2—figure supplement 1 B* | 34a^-/-^;34bc^-/-^;449^+/-^ |
| *Figure 3—figure supplement 1A* | 34a^-/-^;34bc^-/-^;449^+/-^ |
| *Figure 3—figure supplement 1B* | 34a^-/-^;34bc^-/-^;449^+/-^;Hb9::GFP |
| *Figure 3—figure supplement 1C* | 34a^-/-^;34bc^-/-^;449^+/-^ |
| *Figure 3—figure supplement 1D* | 34a^-/-^;34bc^-/-^ |
| *Figure 3—figure supplement 1E* | 34bc^-/-^ |
| *Figure 3—figure supplement 1F* | 34bc^-/-^& 34bc^-/-^;449^+/-^& 34a^+/-^;34bc^-/-^;449^+/-^ |
| *Figure 5—figure supplement 3B~D* | 34bc^-/-^;449^+/-^ & 449^-/-^ |
| *Figure 5—figure supplement 3G~K3* | 34bc^-/-^;449^+/-^& 449^-/-^ |
| *Figure 5—figure supplement 3L* | 34a^-/-^;34bc^+/-^;449^-/-^ |
| *Figure 5—figure supplement 3M* | 34a^-/-^;34bc^+/-^;449^-/-^ & 34a^-/-^;449^-/-^ |
| *Figure 5—figure supplement 4A and 4B* | 34a^-/-^;449^-/-^ |
| *Figure 5—figure supplement 4C* | 34a^+/-^;34bc^+/-^;449^-/-^ & 34a^-/-^;34bc^+/-^;449^-/-^ & 34a^-/-^;449^-/-^ & 34a^-/-^;34bc^-/-^ & 34bc^-/-^ & 449^-/-^ |
| *Figure 5—figure supplement 4D* | 34a^+/-^;34bc^+/-^;449^-/-^ & 34a^-/-^;34bc^-/-^;449^+/-^& 34a^-/-^;34bc^+/-^;449^-/-^& 34a^-/-^;449^-/-^ & 34bc^-/-^ & 449^-/-^ |
| *Figure 5—figure supplement 5A and 5B* | 34a^-/-^;34bc^+/-^;449^-/-^& 34a^-/-^;34bc^-/-^;449^+/-^& 34a^+/-^;34bc^+/-^;449^-/-^ & 34a^-/-^;34bc^-/-^ & 34a^-/-^;449^-/-^ & 34bc^-/-^ & 449^-/-^ |
| *Figure 5—figure supplement 5C and 5D* | 34a^-/-^;34bc^+/-^;449^-/-^& 34bc^+/-^;449^-/-^ & & 34a^-/-^;449^-/-^ & 449^-/-^ |

| **Supplementary file 1g. List of predicted targets of Mir34/449 in Fig. 5B** | | |
| --- | --- | --- |
| **Names of miRNA** | **Total gene number** | **Gene name** |
| MiR34/449-5p  MiR34a-3p  MiR34b/c-3p | 56 | Tmed8 Fgd6 Rfx3 Kctd16 Grin2b Cers6 Cyp2b13 Tcf12 Tbc1d30 Sbk1 Zfp281 Itgb8 Clcn3 Kdm2a Btbd11 Foxp1 Mmab Ndrg1 Zbtb20 Tbck U2af1 Cers4 Hs2st1 Mmp15 Bend4 Slc38a4 Cdc42ep2 Pou2f1 Ctnnd1 Slc8a1 Satb1 Efnb1 Gmfb Dcx Vdr Ubn2 Ccdc85a Fut9 Elf4 Abcf3 CTNND1 Tm9sf3 Eea1 Eef2k POU2F1 Fam63b Rora Slc16a12 Cnot6 Ptpn2 1700025G04Rik Taf4b Onecut2 Rragd Naa50 Itk |
| MiR34/449-5p  MiR34a-3p | 144 | Pkia Syt2 Acsl4 Ppp4r2 Nos1 Grem2 Peg10 Gnaq Klhl31 Rtf1 Stag2 Smad4 Tanc2 Csrnp3 Mapre3 Chmp7 Vat1 Palm2 Scn2b Nr4a2 Ndrg4 Prpf38b Mapt A130010J15Rik Dvl2 Ptpn4 Itpripl1 Scml2 Gjb4 Arhgef12 Rnf44 Dnah3 Myo1c Ppp1r13b Zic5 Slc25a27 Vtcn1 Dcp1a Tspan11 Rnf213 Phf19 Asphd2 2510009E07Rik Crkl Nudt13 Ap5m1 Aldoa Cntnap2 Wdr37 Shoc2 Prox1 Prdm11 Atp2b4 Oxsr1 P2ry2 Slc31a2 Sprn Xylt1 Acbd3 Cln5 Cacna1e Mycn Add2 Tspan7 Osgin2 Rras2 Neto1 Ralgps1 Chtop Map1a Acsl1 Snap25 AF529169 Asb1 Mgat4a Gng12 Thumpd1 Kcnh7 Pkp4 Dock9 Rab21 Foxp2 Ltbp2 Adat3 Tymp Nav1 Ret Myrip Ppm1l Flrt1 Msl2 Dcaf7 Parp8 Foxj2 Chpf2 Ormdl3 St6galnac4 Mtmr9 Igfbp3 Lingo2 Vps33a Yy1 Fbxo30 Rnf165 Snx4 Pet112 C1ql3 Adam22 Nyap2 Gpr158 Cplx2 Nsd1 Rhoj Shisa7 Tbc1d2b Hecw2 Slc6a17 Rap1gds1 Faxc Mllt3 Rnf41 Onecut1 Ric8b Mtcl1 Cdh4 Ten1 Daam1 Snx33 Atxn7l3b Slc25a23 Aff4 Arhgap4 Sec16a Becn1 Chtf8 Sdk2 Sox12 Rragc Tnrc6b Soga1 Nceh1 Cep170b Slc27a4 Hk1 |
| MiR34/449-5p  MiR34b/c-3p | 101 | Gpr64 Cdk6 Arl8b Mdm4 Trim67 Slc31a1 Sptbn2 AW549877 Rims3 Lima1 Arfgap1 Cybrd1 Ttc19 Sh2b3 Plod1 Fbxo41 Spop Lman2l Ralbp1 Fam160a2 D2Wsu81e March5 Prox2 Zfp449 Clock Glra3 Rnf152 Slc7a2 Kcna2 Lgi1 Serpinf2 Fam46a Ank2 Gatad2b Pogz Notch2 Ywhag Sfmbt2 Evi5l Bcl6 Tmcc3 Fndc3a Hnf4a AI597479 Foxn3 Klhl32 Chl1 Arhgap1 Syt1 Akap6 Scn2a1 Zfp641 Numbl Socs4 Prkag1 Ahcyl2 Ppp1r11 Rarg Rgs17 Pou3f3 Cdc25a Cant1 Col12a1 Col4a4 Zcchc17 3110021N24Rik Dgkb Ctdsp2 Slc2a13 Tm7sf3 Gabrb1 Dlgap2 Strn3 Vcpip1 Svop Podxl Nagpa Smim15 Slc2a3 Adipor2 Satb2 Dixdc1 Kcnd3 Jade2 Cpeb2 Zfhx4 Tmem109 Spcs2 Tmem126a Prss12 Suco Hook3 Ubl4 Rufy2 Prex2 1810030O07Rik Map2k3 Rdh11 Elmsan1 Sdccag8 Usp31 |
| MiR34a-3p  MiR34b/c-3p | 949 | Slc22a26 Stxbp5l Trmt5 Mon1b Tmem38b Strbp Gm10220 Ino80d Psd4 Fcrls Ermp1 Fzd5 Snx24 Eltd1 Hscb Dzank1 Zfp113 Cbfb Asap1 Trps1 9230019H11Rik F2rl3 Taf5l Agr3 Rbbp6 Rpl24 Itpripl2 Wdfy1 Rab11b Rasa2 Ssr3 Nudcd3 Cd33 Eif2s3x Il23r Tusc3 Zzz3 Trim24 Rbm25 Ran Fam173b Ccr2 Whsc1 Rps8 Ss18 Pls3 Klk13 Tmem255b AI593442 Actc1 Igfbp4 Luzp2 Nr2c2 5430427O19Rik Lemd3 Nufip2 Ms4a6c Stam D10Bwg1379e Srsf3 Mmp16 Otud6b Fem1c Zmym5 Wrb Tmem47 Dcun1d3 Emc8 Pifo Akap5 Tomm22 Cdca2 Sp4 Rps19 Usp9x Dusp12 Btn1a1 Zyg11b Sim1 Pde4d Ctdspl2 Tspan12 Zfp467 Ptchd4 Dnmt1 Pak3 Agtrap Gm8898 AI464131 Lca5 Kcnv1 Pou4f2 Dyrk1b Slc9a7 Gpr126 Cyp4a14 Bdp1 Klhl24 Acot1 Ept1 Tnfaip8 Brap Gcnt1 St3gal1 Pds5b Itgb3bp Kctd2 Hspa5 Rps6ka6 Tardbp Zscan21 Mul1 Ttl Ammecr1l Capn8 Serp1 Gpr124 Cald1 Zfp74 Alg10b 4930453N24Rik Syt14 Pi4k2b Bco2 Trim44 Tmem207 Myo7a Arrdc3 Slc26a2 Slc35e2 Map3k13 Mapk8 Tgfb2 Cln8 Abcb8 Wasf2 Mbip Tecpr2 Ncr1 Znrf2 Glrx3 Btnl9 Fam71f2 Sfxn1 Pkib Gm12353 Arrb1 Pja2 Thap11 Ppp2r2c Ptprt Bub3 Stx19 AY358078 Lamc3 Nudcd2 Tmem59l Tmem74 Tbc1d16 Plin4 Ppp1r1a Cnksr2 Atr Tmem5 Rgs20 Slc7a11 Chd7 Rad21 Fam149a Zfp704 Hnrnpa0 Msn Btn2a2 Snrnp27 Exoc5 Sftpb Slmap Pex5l Rpl17 Zfp3 Ing1 Setd7 Sema3a Ndfip2 Prr16 Mtmr2 Chn2 Iws1 Cldn25 Kif13a Txndc9 Uba6 Gnat3 B4galt6 H2-T24 Ptpn9 Fign Nkain3 Gm4723 Erp44 Hjurp Syce2 Lancl3 Amd1 Tmem245 Olfr519 Dpf3 Chrm5 Eif4e Il12b Thoc3 1700028P14Rik Rprd1b Ugt2b35 Rps12 Zfp367 Nudcd1 Cntnap5a Pms2 Tmed2 Bmpr2 Kcnk10 Acad12 Tfcp2l1 Lnpep Trub2 Add3 Jazf1 Etv3 Pes1 Ccnd2 Sgcd Ocln Rnf122 Commd2 Katna1 Srek1 Rffl Ism2 Zfp488 Hmcn2 Narf Aspa Asun Espl1 Fgf16 Ptafr Rgp1 Usp29 Slc34a2 Zfp944 Pcdhb18 Hykk Casc5 Ctdsp1 Ebp Iyd Eri1 Otud7b Prkar1a Mob1b Nup35 Zfp141 Samd4 Fubp1 Fam199x Shc4 Snupn Imp4 Ash2l Amd2 Exog Coq10b Fhl5 Ndufaf4 Nell1 Cybb Ccr3 Tmprss11e Kctd4 Zmat3 St8sia6 Ciao1 Rassf8 Lamp2 Sorbs2 Csnk1d Csnk2a1 Arhgap32 Ipp Kcnn3 Trim75 Ctnnbl1 Hps5 Ttc14 Lrrc9 Rdh14 Slc30a7 Trpc1 Zfp26 Rhod Zc3hav1l Chmp4c Srrm4 Itga8 Crk Mapk10 Brdt Hdhd2 Zfp292 Dynll2 Wdr12 Mettl10 Nhlrc3 Rcsd1 1700052N19Rik Ptprq Gm14306 Zfp655 Dbf4 Pcsk2 Wdr89 Ppp1r7 Sec62 Hsf2 Pias2 Cox17 Sftpa1 Asb7 Lypd1 Cd1d2 Zfp607 Ikbkg C77370 Fam222b Pla2r1 Wnt2 Ugt2b34 Nlgn1 Ttc33 Wisp1 Zbtb37 Cask Lect2 Syn3 Mboat2 Galnt13 Farp2 Fxr1 Bcl10 N4bp2 Spin4 Pik3ap1 Gm17567 Ccdc38 Thap4 Nvl Gm14403 Kcnj3 Gabpb2 Hpse2 Nab1 Epha6 Pdk1 Tmod2 Ulk2 Rspo2 Lrrc58 Klf3 Ikzf2 Hspa2 Gdf7 BC094916 Blnk Vav3 Slc35b4 Arhgap18 Mios Ogn Tcf20 Erich5 Gprasp2 Sulf1 Pml Spryd7 Rbm28 Hcn1 Nudt12 Pcmtd1 Lurap1 Chst11 Fam131a Pcdhb5 Ikbkb Cnnm3 Spaca6 Ppl Rpa1 Tal2 Gbp8 Gm4245 Wipf2 Oscp1 Hdx Smoc1 Efr3a Siglec15 Gfod1 Eif4a3 Nkd2 Casd1 Rgs8 Gabbr1 Man1b1 D10Jhu81e Ube2e2 Rreb1 Rasgef1b Ptdss1 Chml Pdik1l BC021785 Olfr354 Phf8 Slitrk5 Nptn Naa16 E130308A19Rik Slc39a14 Ncor1 Traf4 Glrx Pi15 Cyp4f14 Sertad4 Rassf9 Pyurf Esr2 Mars2 Cbln3 Pggt1b Phactr2 Inpp4b Zfp871 Actr8 1200014J11Rik Cenpu Ifnar2 Slc5a3 Kcna1 Apod Slc16a1 Chd1 Cck Runx1t1 Kpna4 Ankfy1 Thrb Upb1 Tox3 Agt Cd93 Abi2 Hist1h3h Dmxl1 Edaradd Zfp873 Gpr165 C2cd4c Celf1 Gm12830 Slc36a2 Cdc27 AW554918 Zfp932 Trim56 Zfp248 Arih1 Gpr17 Tmem33 Ppp1r12b Hist1h3f Plxdc2 Egfr Slc39a8 Lypla1 Mbd5 D16Ertd472e Ppp1r2 Trpc6 Glrb Prpf38a Pacsin2 Napb Ldhb Kcnh5 Mdp1 Zmiz2 6330419J24Rik Dmgdh Itpkb Rbm33 Fbxo48 Rassf6 Usp46 Slc26a7 Clec14a Klhl28 Serpini1 Stk4 Vezt Eif2s1 Gm11444 Plcl1 Rp2h Cytip Nox4 Taf1 Ndel1 Esco1 Scamp1 Mdga2 Thbd Cdh6 Fam135a Hmgcr Mep1a Yipf1 Tbl2 Nkx2-1 Gm9938 Dnajc27 Ccl22 Npr3 Asxl2 Dusp23 Cd38 Mrps17 Arv1 Necab3 Rorb Phldb2 Socs5 Dcun1d5 Flrt2 Atp8a2 Smpdl3a Zfp612 Mettl16 Prkab2 Gm6377 Myt1l Cd8b1 Rgs5 Slco1a1 Fam84a Cnrip1 Map3k9 Ttbk2 Pof1b Adra1a Snx12 Fam198b Fam171b P2ry1 Aak1 Pold3 Ascc3 Ugt2b36 Amer2 2210016F16Rik Rab3ip Fcrla Paqr5 Trim11 Tnfrsf22 Fdft1 Shcbp1 4931406P16Rik Mri1 Ssh2 Cebpb Snn Sec14l5 Rpl37a Brca2 Fam76b Ccin Sytl5 Cdc5l Erich1 Gpm6a Nr1h5 Grb14 Mastl Kcnj1 Cutc Arpc3 C1galt1 Zfp81 Rad18 Ypel2 Tnfrsf13c Naf1 Ltbp1 Slc35a1 Med12l Ttc39b Eif4e3 Antxr2 Acpp Cyp2b10 Rab18 Dclk1 Olfml2a Snap23 Slc4a1ap Zfp825 Gm13889 Mob2 Top2b Ccnt1 Akna Rdx Rab27b Kcnb1 1110038F14Rik Fam120a Zfp39 Impad1 Qsox1 Slc25a42 Fam135b Ptgs1 Wdr20 Atf2 Gpr26 Zfand5 Armc8 Igf2bp1 Pdk4 Tyms Hao1 Tmem178b Rpf2 Sgsm1 Smug1 Myo6 Clip4 Rhobtb3 Sema6d Sfpq Mrpl17 Ildr2 Ctnna3 Gria2 Nckap1 Rasa1 Grb2 Kif26b Psg16 Hddc3 Tsn Ubr5 Ntrk3 1810011O10Rik B3gat2 Gm14440 Mcur1 Olfr60 Ano5 Mup8 Erbb4 Zfp597 Dtnbp1 Pura Olfr1133 St6galnac5 Zfp953 Msi2 Cd2bp2 Nfam1 Trmt11 Ago3 Taz Gm8923 Las1l Ly6a Mpp7 Eif5b Lyrm9 Mctp2 Zscan26 Plet1 Thnsl1 Zfp324 F830016B08Rik Sult1c2 Dpy19l4 Pvrl3 Klhl9 Tmem30a Acot13 Ptrh2 Mad2l1 Fkbp1a Aldh1l1 Ago2 Tsen2 Lactb2 Luc7l2 Cdk18 Gm6086 Aadat Epb4.1 Mphosph9 Stau1 Insr Lmbrd2 Tmx4 Gbp11 Hspa13 Lpp Nov Cep170 Gfm1 Trim71 Kctd1 Zfp879 Zfp930 Cela1 Aqp4 Cnr1 Lacc1 Lhfpl2 Dnajc5 Pard3 Ksr2 Ppp3r1 Kcnq3 S100a7a Irak1 Nras Nrip1 D130040H23Rik Dtnb Ptplad2 Kcnj16 Psip1 Rgl3 Mtap7d3 Suv39h2 Ascc1 Tmem27 Ces1d Gm3646 Mpzl3 Fam217b Cdh12 Kcng3 Bag4 Rasgrp3 Memo1 Fan1 Eddm3b Tns1 Xiap Lmbr1 Cenpm Gmcl1 Pygo1 App Ccdc25 Senp1 Pld1 B3gat1 Tmem41a Btla Fbxl3 Rcn1 Fkbp4 Gpr35 Eif5 Kdm5c Slitrk3 Hnrnpd Csnk1a1 Cmklr1 Nfya Syncrip Sox6 3830408C21Rik Stim2 Thap2 Zfp119a Slco1b2 Gm9776 Zfp92 Kifap3 Fbxw5 Skp2 Abl2 Epc2 Ms4a6b Cycs Tnik Phlpp1 Cdk8 Ikzf3 BC024978 Spata18 Atg4d Ppwd1 Pcdhb20 Pgap1 Etnk1 Cdc73 Fmo2 Gabrg3 Armc10 AA415398 Hrasls Hs6st3 Lpgat1 Ctnnal1 Fdx1 Ap3s2 Slc16a7 Angpt2 Mbd4 Zfp68 Nek10 Rhoh Adcy8 Lrrtm4 Xirp2 Ccdc127 9330159F19Rik Fcho1 Angptl3 Vwa5a Park2 Zfyve16 D1Ertd622e Tet1 Tbx21 Jdp2 Chd5 Tmem64 5430435G22Rik Amer3 Mrpl39 Wtap Glcci1 Nhs Fam210a Siah1a Zfp945 Dcun1d1 Homer2 Gm14288 Zfp831 Heyl Gm10471 Gm4952 Ube2z Cyp2b9 AU022252 Ston1 Otud4 Sepw1 Ola1 Mtmr4 Zfp599 Crispld1 Svip Erich2 Rabgap1l Acap2 Bicd1 Ankrd46 Map7 Isy1 Psmb11 Myocd Prickle1 Ankrd1 Cadm2 D15Ertd621e N4bp3 Cpne5 Zfp286 Neu3 Tmem181a Cd53 1600012H06Rik Kcnj13 4930524B15Rik Tex101 Fem1b Slc25a1 0610009L18Rik Slc5a12 Slc24a2 Inpp1 Igj Stx3 Nxf1 Hist2h3c2 Ccne1 Mamld1 Tcaim Gpr63 Mettl11b Zfp354a Ern1 Ppp6r3 Hnrnpu Sh3rf3 Twsg1 Fgf10 Fbxw2 Frrs1l Fam179a Plekhm3 Kdm5b Arpp19 Lrrn3 Atp13a3 Fam219b Enah Homer1 Tmed5 Pafah1b1 Wbp4 E2f2 Rps6ka5 St6galnac3 Ccsap Nek4 Cldn18 Abcb7 Grsf1 Nt5c1a Ak3 Rag2 Bmp10 Pde6h Ogfrl1 |
| MiR34/449-5p | 365 | Pcdh1 Stk11ip Zdhhc23 Shkbp1 Axl Sox4 Tomm6 Inhbb Fam83h Vamp2 Mpp2 Jag1 Ntn1 Lyst Pfkfb4 Gatsl2 Zdhhc16 Sidt1 Jakmip1 Tgif2 5730455P16Rik Ykt6 Scn1a E2f5 Smc6 Pgm2 Reln Cacng2 9930012K11Rik Fam76a N28178 Ppargc1b Camta1 Fbln5 Galnt7 Dpp3 Pgrmc2 Tmem246 Dmwd Map2k1 5830462I19Rik Mta2 Lgr4 Ralgds Fibcd1 Lrrc28 F2rl2 Gm973 Scai Stk35 Polr3d 2610001J05Rik Atxn7 Hoxa13 Lman1 Plekhg3 Rps6kl1 Trp53inp2 Mtus1 Fgfr1 Rtn4rl1 Tmem255a Fbxl16 Itch Ccne2 Foxg1 Eif2s2 Gabra3 Abcd1 Zdhhc17 Ptprm Ppp2r3a Srpr Rai14 Tmem231 Tusc5 Gpr22 Ephb2 Elmod1 Rnf34 Nrip3 Zfp318 Taf5 Brinp1 Fndc3b Atxn7l3 Sar1a Jmjd1c Slc6a1 Rarb Arid4b Cacna2d2 Pacs1 L3mbtl1 Unc13c E2f3 Glce Hoxa10 Slc37a3 Mmp25 Syvn1 Bnc2 Nsmce4a Frmd4a Lzts3 Zfp316 Mgat5b Synj1 Gm166 Fam126b Tmem184b Gas1 Tom1 Xpo5 Alcam Nono Ldha Ppp1r9b Ptges Sema4c Fam167a Raly Nr1i3 Tbc1d25 Six3 Slc44a2 Itsn1 Ei24 Rab43 Mtmr14 Amer1 Fut8 Atoh1 Kcnk3 Erc1 Zfp553 Fam107a Celf3 Stx17 Gm6133 Stac2 Srr Tmem25 Sept3 Dnm1 Swt1 Atg9a Lhx2 Asic2 Cacnb1 Dgkz Ergic1 Ucn2 Htr2c Gm10401 Arhgap26 Wasf1 Mex3c Ahsa2 Agap2 Prkd1 Sidt2 Crhr1 Ppfia1 Dsn1 Uhrf2 Kcne1l Tmem164 Cntnap1 Sema4b Rims4 Ank3 Gnao1 Nampt Tspan18 Hnrnpa1 Tmem251 Ddx17 Ddn Cntn2 Coro1c Wnt1 Klhdc8a Hspa1b Gnai2 Pid1 Anp32a Tob2 Sgpp1 Ap1b1 Nav3 Camsap1 Crebrf Letmd1 Sync Cbfa2t3 Grid1 Metap1 Smox Rgs1 Snx15 Casp2 Atmin Cemip Klf12 Gab1 Evx2 Grm7 Ppm1a Zer1 Cacnb3 Nrxn2 Ctsa Pip5k1a Ccnd1 Mctp1 Capn5 Sipa1 Cuedc1 Sgta Rps6ka4 Atg4b Hcn3 Coq2 Coro7 Rapgef3 Mpped2 Snx30 Zc3h4 Syn2 Tfdp2 Des Slc35g2 Arx Zfp282 Ascl1 Fat3 Ing5 Brpf3 Ptbp3 Ankrd52 Cenpo Acy1 Shank3 Klf4 Otud7a Zkscan16 Epn2 Myh9 Eno3 Csf1r Tpd52 Fndc8 Kitl Abr Dpysl4 Ctnnd2 Arhgap39 Prrg3 Muc1 Sgsm2 Ralgps2 Ikbip Notch1 Golph3l Slc16a2 Sp2 Tcam1 Elovl6 Slc12a2 Pitpnc1 Ccbl2 Zfp644 Esrra Pdgfrb Zfp593 Neurod2 Mfap4 Plekhf1 Limd2 Tbl1xr1 Ppp1r10 Mxd4 Crtc1 Slco3a1 Trank1 Plcb1 Bcl2 Ncdn Nfe2l1 Fat4 Lef1 Loxl3 Slc30a3 Bcl11b Stat6 Speg Fam210b Adam10 Slc9a9 Sema4f Tmem167b Gpr101 Dph1 Arid4a Met Eml5 Rab11fip4 Ranbp10 Pdzrn3 Serpine1 Mras Ago4 Etl4 Csnk1g1 Gpr85 Ldhd Tppp Pnoc Scamp4 Palm3 Gmnc Rras Pclo Sdhc Rock1 Tmem55a Pdgfra Inpp5k Ppp2r5a Ndst1 Dll1 Fbxo10 Krtcap2 Slc4a7 Plekhh2 Wscd2 Tmem201 Parm1 Mov10l1 Stx5a Zmym4 Lhfpl4 Pea15a Ppp1r16b Gabra4 Ubp1 Foxn2 Vsig10l Magi2 Arhgap36 Gm527 Ap1s2 Cops7b Faim2 Car7 Cnot6l Phf20l1 Usp24 Nrn1 Ptms Vwa5b2 Pan3 Dnajc16 Ppp1r14d |
| MiR34a-3p | 2329 | Rab4a Sergef Sorbs3 Bnip3l Shc2 Ptgr1 Oog4 Rplp2 Sarm1 Dctn3 B3gnt3 Spty2d1 4933440M02Rik Mcm3 Leap2 Mief1 Max Ercc6 Grm4 Gpr37l1 Myo1b Slc25a15 Lzts1 Trim45 Pitpnm3 Pqlc2 Asb14 Scn7a C030046E11Rik Slc35e3 Pnma3 Stt3a Pbx4 Sod2 Nol4l Vangl2 Tnpo1 Dock11 Prps1l3 Pla2g4e Hist2h2be Clec9a Cd209c Gabra1 Fshb Kcnc1 Mmp1b E130309D14Rik Neil2 Nop9 4921524J17Rik Cinp Plp1 Ndufs4 Hbegf Fnbp4 Tor3a Rpp14 Wls Gbe1 Macf1 Casp12 Ctbp2 Rnaset2b Myo5a Ppig Tmem254a Cpd Agpat3 Setbp1 Fbp1 Mpv17l Aif1 Cryl1 Cnpy2 Dclre1b Slc25a33 Ppp2ca Ocrl Mtx3 Sema3e Hlcs Tmem196 Bin3 Zcchc18 Zbtb38 Cdkl2 Pex11a Mybl1 Pm20d1 Bcat1 Pon2 Ankrd49 Lphn2 Plekhb2 Npm1 Gclm Plxna1 Gjd4 Sdk1 Crtac1 Calr Ccdc68 Dmap1 Khdc1a Dpt Snap29 Zhx1 Rdh10 Smok3b Zfhx3 Brwd3 Sfxn2 Kctd15 Klrb1b Paf1 C3ar1 Exosc10 Gm10742 Lrrc32 Avpr1a Htt Rbm12b1 Gars Got1 Atp8b4 Edem1 Gprin3 Spag16 Gm28045 Taf1b Slc5a8 Ppp4r1 Ptgir Dusp7 Gpr146 Zfp935 Gm15217 Zfp760 Mfn1 Cysltr2 Ndufa9 Pag1 Prkci Lpcat3 Camk2n1 Suv420h1 Prepl Sort1 Tmem106a Hoxc10 Nmd3 4931406C07Rik Adamts4 Akr1b8 Cebpg Syt17 Cyhr1 Tmem212 Il2ra Gga3 Yes1 Stk32a Anxa13 Xlr3a Tbc1d4 Srprb Ptpn11 Vmn1r32 Slc5a5 Rexo2 Dhx37 March3 Dll4 Scgb2b24 Afmid Nodal Rnf43 Taf13 Ugt2b1 Cdkn2b Snap47 Pmm2 Nol10 Ctcfl Kmt2e Rad54l2 Prmt6 Pdcl3 Man1a2 Cyp2d40 Hmgxb3 Gmppb Zbtb10 Trim30c Chd2 Lamc1 Slc39a3 Cacnb4 Ints1 Fkbp6 Morc2a 1700102P08Rik Athl1 Ss18l1 Barx1 Josd1 Vwc2l Rnf208 Hlf Vezf1 Txlnb Itga1 Phkb Glra2 Vgll3 Shank2 Arhgef2 Trib2 Avpr2 Poldip2 Cdc42ep3 Asrgl1 Fam193b 9930111J21Rik1 Marveld2 Naip5 Dnajb14 Plcd3 Alg14 Lman2 Jade1 Zfp426 Crtc3 Npas3 Luzp1 Urad Pde8a Atp6v0e2 Srek1ip1 Agxt Stx6 Wdr36 Wif1 Slc38a10 Luc7l3 Krtap10-4 Slc22a6 Adcy1 Rhof Cd200r1 Gm20878 Plxnb1 B3galt1 Plekhj1 Napepld Txnl1 Fyttd1 Fads2 Ccdc122 Frmd8 Gm13225 Eps8 Lrrtm2 Gm13151 Serpina3c Olfr39 Mvk Prdm16 Ncbp1 Stard5 Hamp Kbtbd11 Ddx25 Ccr9 Osmr Slc25a16 Ndrg2 Dapp1 Ryr2 Nacc2 Rab7 Zfp114 Cyb5rl Frg1 Sdc3 2310057M21Rik Fsip1 Gm12166 Wbp2nl Fadd Speer3 Pfkfb2 Gm10176 Dhtkd1 1810037I17Rik Samd7 A530054K11Rik Rnf103 Pcsk5 Mff Cluh Tomm20 Mtor Gm608 Naa25 Nek5 Tmf1 Ubr1 Emc10 Pgm5 Midn Plxna2 Pomgnt2 Golm1 Mfsd7b Nup62 Smarca2 Lsm3 Clmn Mex3a Atf7ip2 Pde1a Prpf39 B9d1 9830147E19Rik Grk4 Ptgr2 Dennd3 E030019B06Rik Kcnq2 Lgi2 Nek7 BC021891 Tec Slc32a1 Git2 Tmem154 Gem Inha Peli2 Ccng2 C1qtnf3 Vmn2r1 Cyp4f13 Grin2a 3110018I06Rik Acbd5 Tgm3 Nme2 Gm10912 Pcgf5 Cnot11 2310005G13Rik Gm6483 Cul4b Cyp1b1 Carhsp1 Kcnj5 Rps2 Klf6 Dennd1b Pigq Slc19a2 F10 Mllt6 Ip6k1 Fcgr1 Rap2b Srgap2 Epha8 Ace3 Crnkl1 Myl10 Pm20d2 Cpe Zfp786 Afap1 Zc3h6 Actr1a Dgke Tmtc3 Gypc Dio3 Gm20390 Rwdd2a Gm9905 Myh4 Zfp276 Iqce Lrrc7 Nmur2 Nupr1 Ndst3 Prkar2a Hnrnpa2b1 Parpbp Narg2 Bbs2 Adat1 Eapp Slc45a4 Plscr2 Hist1h4j Gpr21 Nosip Tubd1 Tacc2 Dicer1 Stk25 Rnf169 Prkcg Usp37 Rem1 Snrpa1 Hmg20a Hivep3 Pomk Zfp616 Pot1b Cmpk1 Umod Stox1 Mmp1a Gm561 Pdia4 Calml4 E430018J23Rik Tnfsf15 Zfp235 Dynlt1c Zfp458 Nat2 Gm21698 Sema3c Chrd Shc3 Oas1a Ptchd2 Gatc Gtf2a2 Stx18 Eif2ak1 Slc44a1 Gjb1 Nhsl2 Slc5a9 Mfn2 Igf1r Cdk9 Chmp1a 6030445D17Rik Gm4894 Snhg11 Negr1 Hmgn2 Reep6 Trit1 Stag1 Tmem254b Mrpl53 Thsd1 Ablim1 Tanc1 Cd164l2 Olfr283 Gm9799 Zfp874a Lin7a Cacng1 Zbtb41 Hist1h1c Srpx2 Proc Ints7 Rnf114 Mrpl12 Pdpk1 Tulp4 Cpped1 Pde5a Sept6 Fgl2 Ccr1 Ctps2 Smurf2 Nefm Ccny Cisd1 Cdh1 Atf6 Trmt2a Kctd3 Stard4 Timm8a1 Cdc42 Dolk Smim20 Fam169a Slc25a3 Gnl3l 4930506M07Rik Sfmbt1 Arglu1 Sumo1 Pou4f1 Shisa3 Ncoa2 Slc16a9 Pax5 Cspp1 Mat1a Dip2b Triap1 Ier5 Cpsf6 Abcg8 Rftn1 Dmbx1 Atp5b Slitrk4 Ifng Il12rb2 Depdc5 Khdc1c Rpgrip1l Vstm2a Hs3st3b1 Arhgap20 Rnase1 Cd59a Pak2 Pde8b Ugt1a7c Jph2 Sgtb Map2k6 Galnt6 4921513D11Rik Btrc Lyz2 Trove2 Txlna Tfap2e Rsl24d1 Tagap Taf1a Gm6685 Ripk4 Zkscan2 Vps37a Mier1 Vasp Rapgef1 Armc4 Nrg4 Abcg1 Lrrc27 Traf6 Cnnm2 Ep400 Prkaa1 Urgcp Pycr1 Gm10610 Micu1 Dgki Lsm14b Chmp6 H2-Ab1 Zmat1 Sh3kbp1 Usp11 Rapgef5 Pde10a Rbp7 Nit1 Myo5c Lrrc57 Ccdc154 Ppil3 Tbc1d9b Nr2f2 Rprd2 Ophn1 Tagln2 Tgds Filip1l Pafah1b3 Fut2 Atp8b2 Spa17 Plekha6 Taf10 Ufd1l Tmem170b Ap2m1 3110002H16Rik Cdcp1 Supt16 Serpina3i Col4a2 Papd5 Sema3f Il4ra Hyal1 Ppib Ccr4 Atxn10 Id4 Dtl Adcy9 Stk10 D3Ertd751e C130060K24Rik Prnd Hist2h4 Dnah10 Epb4.1l2 Cyp2c54 Gzmb Kctd18 Pabpc4l Slc16a3 Chic2 Tubb1 Tmem260 RP24-280O4.9 Dvl1 Gbp9 Oit1 Slc7a13 Rnf185 Sept2 Bcl7c Prkrip1 Gm16039 Pfkfb3 Ptk6 Mcidas Ap2a2 Gm2174 Ddx5 Rit1 Ces2g Fam13c Arf4 Rabgef1 Nacc1 Swap70 Anks1b Nabp2 Zfyve9 Pex26 Tmem135 Plaur Prorsd1 Naa40 Utp14b Pdcd6ip Wdr31 Pced1a Gif Deptor Ict1 Slc4a3 Snapc1 Ptgs2 Prr5l Larp4 Zfp384 Otud3 Cpm Klhl26 Lonp2 Spata13 9130401M01Rik Cd247 U2surp Zfp958 Grk6 Slc25a13 Neto2 Crx Foxk1 Dlc1 Nek8 Atf3 Skap2 Fut11 Lyl1 Herc1 Sulf2 Slc22a23 Dio2 Dgat1 Pkhd1 4732440D04Rik Ripk1 Lin28b Smim7 Pax3 Chst10 Peg12 Tmem161a Gm14124 Rab13 Nfat5 Ostf1 Traf5 Gm6455 Slit3 Icosl Akap7 F930017D23Rik Vat1l Timm22 Npepps Pros1 Adprhl1 Chrnb3 Pnrc2 Klf16 Ccni Lmx1b Zfp623 Amy2a2 Grb7 Cpxm2 Adam19 Fxyd6 Churc1 Ugt1a6a Tm4sf20 Col19a1 Tdp2 Meaf6 Wnt2b Ccrn4l Ubl7 Gm7361 Yipf7 Tmem70 Spns2 Rnase4 Entpd8 Epha3 Efhb Aph1a Tceb3 Gm10681 Gm9989 4932438H23Rik Grin3a D5Ertd579e Surf6 Mterfd2 Clec1a Ctns Syt7 Gnb4 Pbx2 Avl9 Ppp2r5e 4933402N22Rik Slc25a46 Gnptab Hif1an Ddx4 Smad2 Igf2bp3 St7l Prrxl1 Acot3 Lrrc4 Cyp2c38 Cldn8 Elavl4 Slc39a11 Dennd4b Sgip1 Hgsnat Hilpda Hist1h1d Ankrd13a Mak C330018D20Rik Unc80 D630039A03Rik Mapkapk2 Xlr3c Cys1 Exoc7 Gm11110 Gins1 Gpc4 Ptch1 Gabarapl1 Serac1 Zbtb1 Gm906 Tmem68 Ube2f Nfic Mbp Ogfod3 Neurl2 Impdh1 Lpcat1 Fxn C7 Fam168b Sgpp2 Rnf19a Tra2b Nadk2 Cyb5b Egr2 Fam196a Gm7008 Cmpk2 Zfp800 Igfbp1 Nlgn3 Nsun4 Zfp619 Hoxb9 Fam132b Antxr1 Gab3 Evc H3f3b Tmem241 Maml3 Zfp120 Pgp Mmachc Ddhd2 Malsu1 Htr1a Zfp493 Ccdc144b Taf8 BC030500 Alpk1 Baz2a Ifnar1 Hbq1a Ppt1 Nat14 Samm50 Rfc5 Psapl1 Gm19426 Alox5ap Zkscan1 Gm8879 Rsl1d1 Gnai1 Rab29 Fgf15 Apmap Ranbp9 Pafah1b2 Pald1 Nmnat2 B4galt7 Nelfb Grik2 Foxk2 Col4a3 Rit2 Prx Rpl6 Bpnt1 Slc11a1 Specc1 Gm3448 Fchsd2 Mprip Slc23a2 Pcdh10 Btaf1 Ppih Pcmt1 Zfp106 Med6 Rasal2 Mrpl49 Kdelr1 Gins2 Gan Rab11fip2 A930033H14Rik 4931428L18Rik Ptprr Wnt9a Zmat4 Dusp18 Fzr1 Arhgef10 Cby1 Kptn Dhh Rab3d Zscan12 Amy2a4 Pak7 Ttc23 Cyp2d10 Pkd2l2 Hist1h2bk Tmc5 Diablo Xkr6 Cntnap5b Mdn1 Nub1 Ppp1r12a Cyp2j13 Myog Rtkn2 Zfx Trhde Aplf Ugt1a5 Aadacl3 Tecrl Khdrbs2 Phka2 Adamts20 Bora BC031181 Slit1 Arhgap42 1600014C10Rik Ireb2 Pspc1 Klhl3 Gstm4 Ddah1 Tmem184a 3110043O21Rik Wdr96 Nod1 Mrgprh Drd2 Vopp1 Fam212b Mvb12a Angel2 Eda2r Gna13 Rad50 Tacr2 Il17rd Ube2w Timp2 Speer1-ps1 Klhl41 Phf3 Spink5 Vti1a Fgfr2 Mtrf1l Ubr2 Rdh13 Secisbp2l Ptgfrn Dnajc12 Dctn4 Herpud1 Prkcb Hebp1 Usp44 Sowahb Rbm20 Lpar1 Rmdn2 Brix1 Mamstr Sobp Hsf5 Hrct1 Oat Ddx51 Ikbkap Gm14139 Gpd1l Ap2b1 Trim66 Cxcr2 Sec11c Serping1 Fam120c 1700017N19Rik Nabp1 Sell Alkbh7 Asns Slfn8 Gpld1 Atf1 Dhx35 Pde4dip Faah Abca13 Prkca 6530409C15Rik Msl3 Rnf150 Kif5c Dlgap1 Pdp1 Dbx2 Rab14 Unc119b Ugt1a6b Rp1 Dapk1 Dffa Apbb2 R3hdm1 Fbxl20 Mmp14 Frk Fam21 Kank1 Nxph1 Rimkla Hapln2 Rbmx Zcwpw1 Gm4847 Klhl4 Itm2c Mmd2 Birc3 Ankdd1a Kcnab1 Pnpo Acvr1 Sema5a Fam65b Ybey Lmf1 Smad3 Ptpdc1 Ptpn7 Oxsm Hnrnpr Sap30bp Nrxn1 Vrk1 Scara5 Fga Ermn Npc1l1 Cxcl12 Zfp143 Tnfrsf21 6030419C18Rik Maml2 Mtfmt Tmc7 Hist1h2ai Il6ra Ccm2 Entpd3 Olfr653 Slc6a7 D630036H23Rik Fam26d Tirap E330009J07Rik Fam134c Zfp652 Cidec Xrn2 D830039M14Rik Aldh9a1 Tmem50b Larp1b Mlf1 Zfp568 Fgb Kmo Chmp5 Cttn Med29 Lsm4 Oxct1 Tbx18 Gapvd1 Exoc3l2 Adsl Pate4 Col7a1 Rasal1 Olfml1 Ppargc1a Bcl2l11 1700066B19Rik Abca9 Zfp804a Tpp2 Sec63 Sh3bgrl2 Il18rap Zfp595 Mmgt1 Gab2 Ppme1 Osgin1 Lpin1 4933426M11Rik Lims1 Mrgpre 5031439G07Rik Dynlt1a Zfp334 Dnajc6 Slc46a3 Dgcr8 Cacng5 Scaf8 Vcl Cdkl4 Ift20 Elk1 Vstm4 Ywhaq Ccdc170 Slc25a45 Lcor Stat3 Gpr6 Tmem200b Meox1 Exoc6 Aldh2 Steap2 Ywhah N4bp1 Smek2 Pde3a Morn4 Slc30a10 Fbxo31 Ecel1 Rnd1 Cntnap3 Lingo1 Copa Nt5dc3 Spata2 Gldc Lrch4 Ppm1e Ptplb Cyp2j9 Kcnq5 Plcb3 Nfx1 Nova1 Lpxn Sall4 Zbed4 Gp6 Zfp60 Rab1 Ugt1a10 Klhl40 Slc17a6 Ndufc1 Tmem53 Gss 4931440F15Rik Kbtbd7 Bnip2 Ebf3 Zfpm1 Qrich1 Mrpl57 Mrps30 Tmem218 Anapc10 Clpb Mblac1 Slc30a1 1700028K03Rik D3Bwg0562e Nrcam Trip11 Tmprss3 Mpzl1 Ier2 Metrnl Chdh Actr5 Tfec Ythdc1 Jkamp Mynn Rnf24 Ankrd9 Snx31 Gng10 Akr1c14 Lrrc8d Cdh11 Rd3 Pxdn Snrpn Clec16a Olfr330 Il6st Pabpc1l Gm6460 Dsc1 Pdia3 Nudt15 Itga6 2900011O08Rik Fam110b Dpp6 Sord Rhobtb2 Nnmt Bsnd Calu Psmd2 Ppp1r9a Phip Mrrf Tsga10 Tfap2b Hn1l Ric3 Tmtc2 Bptf Kcnk5 Polh Zbtb45 Gpam Hipk3 Fech Mageb3 Yipf5 Ctxn1 Cd4 Mpped1 Wasl Gcat Gm8857 Gyltl1b Rab40b Prpf40a Arl6ip1 Slc13a3 Tmem194b Nxph3 Bet1l Mtfr1 Ehhadh BC005561 Cnih3 Tead1 Ttc37 Prpf6 Glp2r Reep3 Pax9 Ccnj Stx2 Wrap73 Gm17546 Smim14 Samd15 Fam92a Efnb3 Trappc6b Tbl3 Lemd1 Prickle2 Gm5724 Sh3bgrl Cd82 Erap1 Commd8 Ptpn14 Ufm1 Kctd7 Fgf9 Zfp866 Arhgap31 Stard13 Rps6kb1 Pcdhb22 Dhodh Pou6f1 Pcmtd2 Sorcs1 5830473C10Rik Acadsb Polr3g Syce1 Ppp1cb Pln Nfkbie Map3k7cl Ubiad1 Naa11 Dync1li2 Tmem66 Rlbp1 Crem Nckap5 Chst15 Actg1 Ugt1a1 Ikzf1 Acvr2a Dcdc2a Tmem119 Crcp Gosr2 Sft2d1 Zfp11 Prlr Anapc15 Slc2a9 Gm20708 Zpbp Rsf1 Nup214 Slc8a2 Krt75 Haus6 Hoxd9 Ndufb11 Lhx4 Rab8b Uggt1 Idnk Prrc1 Kif1c 8030423J24Rik Cep97 Gpr132 Aldoc Atg2a Nedd1 Ankrd63 Cdk15 Hn1 Emx2 Smap1 C87436 Pptc7 Arl13b Xrcc6bp1 Naa60 Aplp2 Agl Rassf3 Liph Nxpe5 Sec1 BC003965 S100pbp Med19 Tcte2 Sdpr 4930544L04Rik Zbtb33 Os9 Tmppe Pdlim5 Eid1 Paxbp1 Pwp2 Adipor1 Cbx5 Gm7347 9130019O22Rik Ugt1a8 N6amt2 Ube3c Erv3 Ttpa Mterf1b Rgs22 Ippk D10Wsu102e Mrps14 Senp5 Dlg2 Dgcr14 Slc12a1 Il19 Apoo Drg1 Msantd1 Lrrc42 Golga2 Grm1 Ugt1a2 Gimap5 Lrrc56 Ppfia2 Camsap2 Mpp6 Ids Tcp11 Tgm1 Smndc1 Syt10 Ctu1 Rab30 Soat1 Phlpp2 B4galt5 Smg1 Aph1c Wbp1l Arpc1b Cnpy1 Cdk5rap3 Sp1 Foxred1 Sult1b1 Agbl5 Kbtbd13 Ccdc96 Prmt3 Gen1 1110008L16Rik Slc38a11 Ccl6 Tgfb1 Slc25a10 Tff3 Prcp Maff Gm16314 Pdzd8 Chn1 Gm9845 Cd160 Slc4a9 9130409I23Rik Slc10a5 Kcnh2 Obfc1 2610528J11Rik Tub Rmnd1 Rad23b Tet3 Fbxo7 S1pr2 Slc5a1 Colgalt1 Ndc1 Mobp Sf3a1 Sf1 Gm20503 Galm Tiprl Polg A530053G22Rik Cyb5d1 Gpkow Nrp2 Naa35 Kcns2 Peo1 Phf20 Grap2 Slc15a5 Acot9 Glp1r Zfp87 Bcl2l13 Zfyve27 Clns1a Cldn2 Thap3 Cyp2d37-ps A230046K03Rik Mcc Tbc1d19 Clec4a2 Rnf138 Zbtb46 Glyctk Ing2 Med1 Sall3 Mc2r Nebl Gm21092 Cdan1 Sectm1b Mcoln2 Tmem18 Rnf183 Rpusd2 Cacul1 Aldh1a1 Dstyk Aldh1a7 Vsx1 Gm8890 Gm15401 Frmd3 Cyld Lats2 Lrp1b Slc1a2 Clec2d Cox5a Cabyr Sec24c Dapk3 Irf4 Gm4450 Tbc1d8b Nell2 Cacng8 Sucla2 Fam115a Fam228b Dnhd1 Wnt7b Arfgap3 Stac Zfp217 Nupl1 St3gal6 Cyp2a5 Fkbp5 Rgs7bp Prkrir Dhx33 Fam19a4 Col14a1 Prss41 Smok3a Ccr5 Ces1f Qk Dhx8 Suclg2 Ppp1r3b 1700101E01Rik Pus1 Rgag4 Gata5 Armc3 Trp53bp1 Smarcc1 Erlin2 Larp4b Vmn1r65 Tmem256 Plcb2 Lfng Rbm38 Pcdhb10 Ppp6c Rbm18 Dnajc22 Plekhf2 Gm10309 Mup7 Zzef1 Smarca5 Atp10a Ankrd34c Parp16 Nphs2 Ttc29 Pou3f4 Ets1 Wdr52 Smcr8 Ccdc59 Ahsa1 Gpr133 Ackr2 Ninj1 Rps3a1 Rnf217 Dennd4a Mknk1 Smim4 Dnah5 Nxpe3 Atg2b Asb16 Chst2 Bhmt2 Aass Zfp955b Adam11 Mipep Ankef1 Blcap Lifr Dctn1 Zfp109 Stoml1 Paip2b Tenm3 Atp13a5 Furin Tmem203 Alx4 Sp110 Plxnc1 1110032A03Rik Pllp Ccdc158 Trim40 Mn1 Ddx1 Pddc1 Skp1a Fgf12 Abca5 Stmn2 Tdo2 Ccdc63 Chrna3 Bloc1s5 Rc3h2 Tifab Rps6ka3 Wibg Fto Fbxw8 Uqcrq Zfp420 Mpdz Gm14221 Kcnb2 Rilpl1 Ikzf4 Inhbe Usp7 Tctn1 Vmn2r57 Bcat2 Zfp12 Gm14569 Kdm4a Hif3a Zfp654 Il17b Ephb3 Rap1gap2 Tusc2 Plekha8 Epb4.1l4b Mgat5 4930415F15Rik Pde2a Tac1 Depdc1a Osbpl6 Pogk Calm3 Ncbp2 Amy2a3 Coq3 Kbtbd12 Pdgfc Rfx7 Cyp4f37 Pip4k2c Exoc8 Git1 Acsl6 Slc37a2 Smad1 Vmn1r44 Cat BC022687 Pnp Birc5 Mro Gm10447 Nat8l Pfn4 Klhl23 Tbr1 Dzip1l Cbx4 Apol10b Hs3st3a1 Syt11 Ntn3 Slc6a15 Stx11 Ide Cbx6 Il15 Acp2 Zfp365 Ugt1a9 Lyrm4 Kcnj15 Slc9a6 Cfhr1 Cdx4 Hmgcs2 Hsd3b4 Gm10518 Krtap12-1 Pnp2 Hcar1 Cntn3 Hoxa1 1110057K04Rik Zfp160 Mtpn Sugt1 Rbp4 A830018L16Rik Etv1 Nfatc4 Vps26a Gcc2 Hspa12a Cdk2ap2 Mmp28 Rngtt Elavl1 9130230L23Rik Cd200 Supt4a Srpk2 Gm15440 Kirrel Arcn1 Hrsp12 Atp2a3 Gm6356 Mpv17 BC030336 AI314180 Hypk Gpatch2l Slc45a3 Ccdc85c Slc6a5 Sh3bp5l Stard8 Zfp148 Clic5 2610015P09Rik Fzd3 Slc16a6 Olfr325 Otc Utp15 Pepd mt-Nd6 G0s2 Ighmbp2 Pdpr Wrn Nme1 Col11a1 Tmem129 Chchd10 Sos2 Epha7 Uqcc1 Pah Caskin1 Wdr54 Ticam1 Derl1 Rnf145 Gm5861 Gulp1 Kif20a Sprr2e Ap1ar Hal Nup62cl Dmrta1 Mccc1 Rhou Gbp4 Vwde Gas2l3 Erp29 4932431P20Rik Hectd1 Gpr123 Gm17174 Ccl24 Fam192a Ip6k2 Neu4 Intu Zfp345 Dnajc30 Gm3625 Dpep2 Gpn1 Tmem156 Xlr3b Gm9804 Mcmbp Olfr433 Cox19 Tlcd2 Zxdb Timm10b Pex10 Stub1 4632415L05Rik Litaf Has3 Fzd7 Abcd2 Tbc1d10b Ins2 Adnp2 Robo1 Parg Gm6432 L3hypdh Otx2 Mthfsl Atp5s Wdr26 Plxdc1 Stk38 Gnai3 Tgoln1 2810459M11Rik Ercc2 Ces3a Llgl1 5430421N21Rik Gm17296 Sbk2 Acot11 Pknox2 Slc17a9 Sned1 Gfra1 Pom121 Fmo5 Apln Dnajb2 Sox11 4933427D14Rik Lrp11 Hpcal4 Maoa Pikfyve Abcc5 Shroom4 Ado D430019H16Rik Vwa2 Actb Itga4 Gm9797 5031410I06Rik Fbxo40 Csf1 Fam114a1 Zfp93 Uaca Tle4 Zfp395 Fam189a1 Tpm3-rs7 Dync2h1 Col4a3bp Rbl2 Amacr Iqgap3 Rasa4 Csf2ra Tmem254c Ndufa4 Tpgs2 Osbpl10 Dock1 Rcbtb2 Tbc1d15 Rwdd3 Kank2 Zbtb42 Rbfox2 Abra Mab21l1 Usp15 Rapgef2 Rps15a Capn13 Emp1 Psmd1 Zfp574 AI987944 Adamts14 Scube3 3110062M04Rik Dlg4 Gm1123 Vps37d Thrap3 Pgpep1l Krtap31-2 Gpr180 Zfp937 Mapk9 Galnt10 Atp6v1a Cep250 Smc3 Wdr72 Slamf7 Abo Ube2u 2310045N01Rik Dock8 Gm7120 Fbxw17 Vsx2 Cul3 Dhx15 Gm6465 Ndufb5 Fah Ndnf Tmem74b Hephl1 Cap2 Pabpc5 Gm15557 Lsamp Zfp35 Cr1l 4933421I07Rik Btnl2 Msantd2 Lipm Enpp1 Speer4a Ifi203 Trmt2b Zfp280c Wfdc8 Hao2 Rbak Hap1 Rab26 Pign Rb1cc1 Zfp329 Cog6 Dido1 Gabrb3 Cst10 Klri1 Pgbd1 1700016C15Rik Tmem107 Eif4ebp1 Ldlrap1 Mia3 Srsf5 Sema3b Necab1 Hmga1-rs1 Sgol1 Mib1 Plch2 Olfr119 Optn Tnnc1 Mb21d2 Irs1 Mcpt4 Efcab11 Ssx2ip St8sia1 Hsd17b1 Clstn1 Skida1 Gtf2e1 Cav1 1700017B05Rik 1810011H11Rik Fem1a Eny2 Nr2f6 Akap11 Ctsc Dpy19l3 Elovl4 Speer2 Exoc6b Atp6v0b Thada Hs6st2 Eif5a2 Tmem45b Ttn Ciapin1 Asb13 Tusc1 H2-Ke6 Higd1a 2210016L21Rik 2010315B03Rik Tfam Slc7a1 Alg6 Gemin2 Acp1 Ifitm2 Myoz3 Atg14 Yod1 Ptprc Cdh26 Cdk5r1 Bcl2l2 Klhl42 Sbno1 Scp2d1 Igf1 Zfp940 Tmem209 Ptpre Lrrn4 Rnf216 Fam180a Zbtb49 Esrp2 Hnmt Gm20425 Stxbp6 2310079G19Rik Mllt11 Gpr107 Tomm70a Itgad Rgs9bp Ftcd Mga Ces3b Adamts15 Smim1 Zfand3 Fzd8 Tgfbr3 Wscd1 Mob3c Dpm2 Mxi1 Asb4 S100a11 Scrn1 Tmem26 Mbl1 Acot6 Kiss1r Galk2 Zbtb25 Ddx31 Pip4k2a Nudt16 Cdk11b Llgl2 Ppp2r1b Rab5b Phf21a Pcdhb12 Slc25a24 Rxfp1 Med22 Itfg1 Mfap5 Tmem261 Lss Cmtm6 Ankrd34a Zfp157 Clta Myo5b Cuedc2 Cript Fscn1 Cnot3 Rgs4 Map3k1 Lst1 Cab39 Chodl Cyb5r2 Ebf4 Gria4 Pou3f2 Efemp2 Sept11 Mill2 Scoc Smim17 Urah Zfp422 Lypd6b Tex261 2010109I03Rik Oasl2 Golga1 Ywhaz Igfn1 Cdk2 Wnt4 Atad1 Paqr9 Derl2 Armcx3 Kbtbd2 Cdc6 Tars Adcyap1r1 Gclc Sh3tc2 Acer1 Pdxdc1 Slc25a12 Ptrf Zfp263 Cacna2d4 Adamts1 Spopl Ces1c Trpv3 Snrnp35 Abcc3 Asxl1 Rnaseh2a Fktn Sapcd2 Adi1 Mroh7 Wwp2 Gc Fezf1 Itpkc Ubtd2 Gm16485 Setd5 Heatr1 Kcnk4 Hnrnpab Psmd8 BC030499 Lrrc14b G3bp2 Msrb1 Pcm1 Zfp697 Kcnmb1 Dmd Gm9970 Synm Olfr701 Pgk1 Abcf1 Lix1l Zbtb44 Mettl6 Slc7a14 Lzic Spry1 Primpol Gda Msantd4 Lynx1 Specc1l Loxl2 Sc5d Nup160 Hdac4 Trnp1 Ttc9 Canx Pde4a Chpf Trmt13 Msantd3 Gprc5a Eif4e1b Trim16 Pik3ip1 Cln6 Polr3a Pcdh19 Dlst Efcab2 Irx6 Hapln1 Scn1b Kif11 Bves Pigm Obp2b Rbm27 Pvalb Unc5c Pfn2 Best3 Gtf2h3 Adck4 Mau2 Lpin2 Camk2g D630045J12Rik Ifit2 Cbx2 Pdia6 Ddx47 Hesx1 Stk39 Gramd1b Timm17b Gdf2 Ccdc137 Adm Ccdc88b Arhgef9 Srgap1 2010012O05Rik A1cf Cstf1 Zmpste24 Caln1 Sncaip Stk32b Cxcl16 Ssh1 Cbfa2t2 Casq2 Cttnbp2nl Scyl2 Klhl38 Gria3 Dgat2 Kcnj9 Iars2 Ramp3 Ubl5 Tmem39a Echs1 Sult1d1 Gm5150 Igsf3 Rrm1 Incenp P2rx5 Nr1d2 Mlxip Zfp850 Steap4 Ppp2r4 Zfp746 Snx11 Tgfbrap1 Klrb1f Psd3 Klhl7 0610010F05Rik Lipg Myoz1 Cbl Hsd11b2 Dcxr Dppa2 Il10ra Fblim1 Usp14 Usf2 3110035E14Rik Gm10118 Dcaf5 Scrn3 Emx1 Ggt6 Zfp397 Aars Tmem97 Chic1 Nuf2 Srsf4 Spire1 Ccdc184 Lrrk2 Zfp174 Thap1 Chrdl1 Cyb561d1 Lmtk3 Usp48 Zcchc14 K230010J24Rik Clec4a1 Dhx36 Ccr6 Sox7 2010111I01Rik Tln2 Sec23ip Gosr1 Vmac |
| MiR34b/c-3p | 2121 | Dhx9 Ube2l3 Dcun1d2 Gm14444 Scaf11 Pex19 Bcl7a Gm14419 Rybp Dnajc21 Lrig2 Gm10377 Mthfs Fam187b Cept1 Gm9875 Psmg2 Ntrk2 Dhrs7b Zfp58 Ifna12 Tlr9 Ggh Htr5a Lrat Rnf115 4930539E08Rik 1700019D03Rik Ddx52 Sspn Mex3b Setdb2 Peli3 Pgap2 Clec2h Stk24 Oip5 Rab9b Irs4 Nrep Bbx Cnot4 Immp2l Nmt1 Arhgap29 Clec4g Dnah17 Nup210 BC030867 C1rl Slc6a18 Camk4 Fam81a Olfr77 Klb Zc3h12b l7Rn6 Sufu 5330417C22Rik 2810004N23Rik Sorl1 Ly6e Ermap Sec13 Slc6a20b Tmcc1 Selt Gm4980 Gpr156 C1qtnf7 Ccdc134 Sucnr1 4933436I01Rik Gm12184 Magi1 Hs3st1 Fsbp Baz1a Lonrf3 Gprc5b Rab11fip3 Zfp558 Tysnd1 Eif3h Mas1 Med14 A330021E22Rik Krtap3-3 Eed Tmem106b Xk Lipk Syt9 Brms1l Lrrc59 Zfp472 Tktl2 Akr1c6 Hist1h1a Thumpd2 Gm3543 Lamc2 Ttll6 Ott Nipal1 Sfr1 Vipr1 Card6 Rnf168 Pir H2-Q10 Cox7b Gm8279 P2ry4 Dennd2d Cadm1 Fcgr4 Lrrc18 Mtus2 Rgmb Rfwd2 Stxbp5 Abcc9 Mt1 Dpy19l1 Prom1 2210018M11Rik Runx2 Sfrp1 Upf2 Dhrs13 Sdf4 Ptprz1 Harbi1 Pcdh18 Gm3486 Map2 Plagl2 Tctn3 Fancl Spred1 She Fkbp15 4930578I06Rik Herpud2 Cd46 Ext1 Klrk1 Mr1 Lrrc20 Lsm10 Trafd1 Osbpl1a Alas1 Ghr Gm1564 Olfr443-ps1 Gpc6 Sgk3 Mipol1 1700001F09Rik Cpt1a Elmo1 Duox1 Rab2b F2r Chp2 March11 Bicc1 Tuba1b Atrnl1 1700048O20Rik Ahcy Ghitm Sms Bmp2k Elavl2 Kcna4 Esp31 Kdm5d Kcnq1 Creb3l2 Abca6 Vps39 Stk38l Ankib1 Ngf Evi2a Rbpj Fbxo45 Gemin8 Ece1 Cpn2 Samhd1 Gm2026 Nup153 Mfsd2a Nhlrc1 Glis2 Tmem150b Zfp236 Pank1 Gm2042 Smarca1 Fam19a3 Trmt1l Dnase2b Sos1 4932411N23Rik Dcp1b Arl5b Zdhhc9 Nxt2 Zfand1 Foxp3 Mroh6 Gm5475 Arl14epl Atp11a Tmem205 Dmpk Fam185a Irak3 Usb1 Mpc1 Ell2 Meis2 Tnfaip8l3 Ifnlr1 Robo2 Cmtm4 Mtr Acsm1 Cacna2d1 Rmi1 Fnip2 March6 Mrpl13 Abi3bp Nktr Grpr Fpgt Tubb3 Wdr92 Msr1 Tmem179 Aasdh Ocstamp Morf4l1 Sf3b4 Crb1 D330045A20Rik Trabd2b Bhlhe22 Uprt Agps 4932438A13Rik Epha5 Slc17a2 Tldc1 Nhp2 Pus7l Cd226 Pphln1 Zfp609 Mettl2 Sdr9c7 Gm4724 Rrp15 Opn1sw Pyhin1 Fancf Lamtor3 Cxcl9 Efna5 Asf1a Tdh Svep1 Serpinb1a Zfp668 Ms4a8a Cand1 Popdc3 Hgd Gm4181 Col25a1 Zfp398 Slc12a6 Apopt1 Shisa6 Calb1 C230062I16Rik Dusp27 Elovl2 Zbtb7c Fzd2 4833424O15Rik Lin54 Metap2 Cryz Mgat4c Gucy2c Rnf144b AI467606 St6gal2 Suz12 Lias Pdha1 Bcap31 Cd2ap Glul Scara3 Tollip Panx1 Cyb561 Rab3b Mpp3 H6pd Cox7a2 Sec61g Jmy Rps20 Themis Ripply3 Gspt2 Crot Ccdc163 Ifltd1 Nudt10 Gm15107 Ereg Sept7 Pld5 Gpr82 1810009A15Rik Gm5900 Zfp933 Mecp2 Kctd12b Rnf214 Plb1 Zfp202 BC026585 Gm11595 Ankrd12 Sf3b1 Xpo7 Cyp4f16 Gm14418 2410004P03Rik Rhbdl3 Gbp6 Mical3 Mrs2 Dnaic2 Nt5e Tnfsf18 Trim36 Msx3 Fam136a Epb4.1l5 Thoc6 Pcdh17 2210418O10Rik Rhbdd2 Nfrkb Gdpd1 Zfp518b Synj2bp Taok2 Slc36a4 Rsrc2 Esyt2 Dennd6a Zbtb21 Map4 Six4 Tmem38a Chrna7 Abhd13 Cacna1c Rps7 Rpa3 Plagl1 Kif2a Pola1 B3galnt1 Aga Slc6a11 Eif3b Vip Ptdss2 AU021092 3632451O06Rik Pdyn Naalad2 Esrrb Zfp629 Baz1b Rbp1 Paics Irgm1 Pot1a Gorab Umps Krtap6-5 Lancl1 Ccdc15 Slx1b Spock1 Tmem178 Simc1 Gpr119 Ndufaf6 Zfp661 Mlh3 Rsl1 Zfp799 Slc7a6os Oprk1 2700060E02Rik Fam49a Zmat2 Chm Tarm1 Dennd2c P4ha2 Tgm2 Nsun3 Kdm7a 1110002E22Rik Heatr2 Zfyve20 Ccar1 Tmem45a Lrp12 Cox16 Fbxo3 Gtpbp2 Slc25a35 Chid1 Clic4 Rspo4 S100b Galntl6 Pdgfb Wnt3 Dnaja2 Igf2 Slc13a1 Snrnp70 Sybu Cyb5r4 Zmynd8 Fam193a Ap5b1 Edrf1 Mef2c Gm20815 Coq7 Dusp8 Sesn3 Gm14399 Zranb3 Tsku Tmem198b Ankrd13c Bmp8b Ptx4 Trim5 Ppif Pik3cg Zdhhc21 Timd2 Kbtbd3 Itgbl1 Sestd1 Fcho2 Fam204a Thoc1 Ston2 Uchl5 9230110F15Rik Gm14295 Mrpl32 Ubac1 Lamp3 Ppp2r5d Dok4 Sec23a Rap2a Gm5819 Ephx4 A530064D06Rik Sgk2 Taok1 Cbs Wdr48 Reep1 Cerkl Slc26a3 Sec11a Gramd2 Shq1 AI429214 Ccdc115 Api5 Akr1c20 Gm14434 Klhl11 Inhba Far1 Pbx1 Zufsp Sh2d7 Son Pawr Trim12c Piezo2 Cadps2 Gm13152 Gm14410 Ptpn1 4930516K23Rik Gm14420 Parp11 Ehd2 Lrrc19 Sebox Appl1 Ube2j1 Has2 Mycs Cpne3 Fhod3 Ndrg3 Tigd2 Lrpap1 Gm156 Ubxn7 Chrnb4 Fam19a1 Acsm2 Mki67 Brip1 Pcdh11x Tnc Srd5a2 Slc16a14 Mat2a Kl Nipsnap3a Impact Pet2 Nxt1 Il16 Spic Col22a1 Hdhd3 Cd79a Elac1 Knop1 Grm3 Prpf4b Marveld3 Csnk1e Entpd5 Cep350 Spata6 Dnaaf2 N4bp2l2 Pde11a Dao Zbtb9 Zfp706 Gm2381 Slc26a9 Sec14l3 Hint3 Ncapg2 Flt3l Mmp2 Plin3 Cyp7b1 Ssr1 Gm4788 Serpinb9g Rassf2 Strap Ikbke Slc13a5 Iqsec3 Gpr179 Rab11fip1 Prkar2b Fuca2 Sdad1 Rab3c Dld Bcr Ggct Plscr3 Sugp2 Tab1 Ift57 Pla2g16 Hdac9 Rtp1 Rfx5 Trpm6 Taf7 Gucy1a2 Pctp 1810041L15Rik Lrtm1 Mamdc2 Syne3 Nkiras1 Zfp606 Tmem183a Sla Epb4.1l4a Ttc32 Zfp251 Olfr1029 Csta1 Itpr1 Pydc4 Fam216b Pacrgl Gm6614 Map9 Sprr2a1 Mdh2 Ntng1 Serpinb9f Wbscr17 Catsper2 Prdx2 Nat10 Slc12a7 Rdh7 Atp9a Gm4944 Mcl1 Mrpl41 Azgp1 Zfp622 1700019N19Rik Cyp51 Cdk20 Irgm2 Sertm1 Nol3 Clip3 Spata4 Gcgr Mospd2 Rmdn1 BC053393 Ptcd3 Myl4 Akap10 Smcp F9 Xpo6 Gm4737 Dhrs4 Pla2g4c Mvb12b Tpmt Ankub1 Gm15114 Adamts12 Vmn2r34 Hoxd13 Tmx3 Agap1 Ints12 Man2a1 Tnfaip8l1 Ces2a Xpr1 Dpp8 Traf2 Lgmn Aco1 F13a1 Taldo1 Zfp770 Cep78 Rab23 Eif3a Gm9887 Ppp1r15a Ankfn1 Fam133b Bhlhe23 Otop1 Htr6 Atp11c Sft2d3 Arfip1 Heatr5b Actr10 Smarcc2 Chrm2 Kcnf1 Uts2b Gtf3c3 Inip Tmc6 Ptbp2 Hist1h2ah Trim25 Upk3b Baat Tbc1d1 Rprd1a Tnxb Kdelc1 Adamts6 Samd5 Mettl21c Ap3m2 St8sia4 AA986860 Kctd6 Cblc Pdlim1 Ddr2 Xrn1 Urb2 Pum2 Ube2o Mzt1 Bmp5 Mfge8 Prkd3 Tssc1 Bbox1 Elmo2 Mast2 Serpinb10 Ccdc112 Gid8 Slc7a12 Rnf125 Kif3a Ntsr2 Lrch1 Pdcd5 Eya4 B3galt2 Slc6a14 Hmgb1 Rnf149 Rufy3 Ankrd44 Oas3 Gdap2 Xlr4c Dcbld2 Tmem163 Prim1 Sumo2 Morc4 Gpr39 Lilra5 Mplkip Spib Vmn2r45 Erbb3 Polr3k Tyw5 Abca1 Tdrkh Lrrc61 Plec Myo3b Tmem175 Olfr1164 Glyat Raph1 Perm1 Mc1r Mecom Ltc4s Caps2 Ormdl1 Acvr1c Lair1 Chpt1 Arhgef10l 1810013L24Rik Rasef Cd36 Tmem126b Mrm1 Spry4 Prg4 Cngb3 Epsti1 Rsbn1 Spcs3 Bckdk Chchd4 Nsl1 Barhl2 Ipo7 1810022K09Rik Eps15 Ccs Trim61 Rev1 Krtap9-1 Strn Agfg1 Ppat Tmem125 Tax1bp3 Mbnl3 Gm10302 Lrrc8b Speer4b Zc3h7b Hivep1 0610040J01Rik Gata3 4921528I07Rik Lphn1 Serpinb9e Krcc1 Zdhhc15 Arel1 Olfm4 Mei4 B430305J03Rik Atp8b1 Spice1 Ndufb9 Igfbp7 Plekhs1 Actrt2 Pla2g4d Acox1 Klhdc10 Pdzd11 Deb1 Abca8b Ptpra Pax8 Rad9b Ccdc14 Il1f5 Wash Reps2 Vps36 Usp1 Vmn2r40 Zfp317 Wdr18 Set Cep120 Bzw1 Wdfy2 Mrpl15 Cr2 Iqub Muc15 Pycr2 Chil3 Serbp1 Ano3 Ivns1abp Sepsecs Dnah8 Rasgrp1 Pradc1 Myh2 Slc2a10 Tpm4 Naa15 St13 Cnnm4 Cse1l Haus8 Gm9881 Cks1b Ppil6 Bnip3 Rpgr Cdkl3 Gm15800 Fbxl7 Slc39a7 Pcca Usp40 Necap2 Sart1 Dach1 Olfr1034 Nmt2 Ccdc82 Acnat2 Trim27 Lcn4 Gm14443 Frem2 Vamp3 Tmsb4x Arhgap15 Mtf2 Dyrk2 Col5a2 Lin52 Ptp4a1 Eif2b5 Zfp84 Slc25a36 Vta1 Olfr78 Vbp1 Rrp7a Plxna4 Marcksl1 Atp2c1 S100a8 Sptlc1 Cd40 Alx1 Sbk3 Clmp Cyp3a11 F930015N05Rik Zbtb8os Otulin Slc33a1 Clstn2 Pde1c Ddx19b Etnppl Wee2 Zfp689 Aldh4a1 Ppp3cb Usp6nl Taf9 Ttll10 BC005624 Fam150a Slco5a1 Nek9 Slc7a4 Aen Rplp1 Psma1 Cylc1 Papolg Ubfd1 Gm15097 Snx25 Nfe2 Fads6 Zfml Ccdc6 Pex14 Spaca1 3110070M22Rik Ly6i Hgf Tmem136 Vash1 Ncf1 Cyp2j5 Gm10032 Smim13 Eif4a2 4930579G24Rik Aoah Ltn1 Nudt21 F8a 2810021J22Rik Rinl Ccbe1 Rnf128 Zfp719 Elp4 Pnisr Dkk2 Cbwd1 Gm2007 Kif3b Zfp459 Ino80c Tbcc Ncam2 4632428N05Rik Tceal1 Emp2 Lpl Hmga2 Trp53i11 Armc1 Itgb5 Scube1 Zfp648 Per1 Apip Nucb2 Izumo3 Cpeb3 Kdelc2 Gm20498 Caprin2 Klhdc2 4932411E22Rik Tfr2 Apol11b Ap3m1 Rnls Zc3h10 Tia1 Siae Slc25a37 Scnn1b Zc2hc1a Irak4 Ccdc80 Nwd1 Aox2 Gm14326 Elf1 Wdr44 Ttf1 Ipmk Rgag1 Stox2 Psmd4 Aldob Arsb Cog2 Siah2 Repin1 Zfp827 Cd1d1 Ugt2b37 Pef1 Vmn1r180 Shisa9 Scel Pin1 Gng5 Slc17a4 Rcor1 Gm14305 Il7 4930538K18Rik Cdk12 Cep135 Htr1b C87977 Slu7 Hist1h4h Slx4ip Tes Slc52a2 Tmem43 Klra10 Aagab Mphosph6 Six6 Gm14698 Lrrk1 Sh2d4b Mmp17 Ces1h Dnaja1 Ganab Gm11397 Draxin D130043K22Rik Il22ra2 Gm6710 Tmem63c Slc11a2 Kat2b Mfsd1 Mut Cdh7 Maea Nrarp Clvs2 Gm4631 Pik3c2a Ccdc147 Pdp2 Scrib Tmed10 Ptchd1 Tmem19 Rif1 Cldn1 AU015836 Dysf Astn1 Cyp4x1 Olfm3 Ppfibp1 Cenpn Edf1 Parp1 Gm10439 Prss16 Dcc Kpna1 Cep57l1 Tnfaip1 Plcxd2 Cetn1 Gm5868 Frem1 Fam115c Setd3 Zfp111 Yaf2 Bmp3 Arf2 Srl Mtmr12 March7 Invs Ttc39c Zfp942 Vwa9 Psmf1 Cdon Gm21743 Dirc2 Gtf2b Zfyve19 Diap2 Map1lc3b Gm28048 Exosc1 Kcnj14 Orc3 Gm14308 Ganc Zcchc24 Efcab8 Arl14ep Gm10941 Hmbox1 Pdzd9 Agpat9 Zfp551 Sv2c Gm4951 Rbms2 Ube2e3 Gm15127 Mapk1ip1l Cdkn3 Ptprg Neurod4 Neurod1 Slc9a2 Gcnt4 Siglece Adamts7 Zeb2 Zfp128 Gdpgp1 Npbwr1 Sprr2k Ipo5 Vmn2r43 Ttll4 Zfp738 Pla2g7 Zfp182 Oraov1 Gpbp1 Cstf2t Fam101b Nrg1 Sp8 Kansl1 Mrgprb2 Zfp322a Pebp1 Eogt Rimklb Epb4.2 Galr1 B230219D22Rik Dsg1a Hnrnph1 Kif3c Zfp839 Gria1 Srp54a Zic1 Vapb 5730596B20Rik Mtm1 Mrpl14 2010107E04Rik Ypel5 Fos Cisd2 Mbd3l2 Hey1 Polr1e Atp1b1 Kcnk12 Gm19684 C6orf183 Lmod1 Ankle1 Rab17 Eif4g2 Idh2 Agtr2 Mertk A630007B06Rik Zfp445 Ldlrad4 Stam2 Mob3b Gm10369 Dpp10 Dusp22 Fbxl18 Ogfod1 Foxf2 Mgst3 Btg4 Rela Npsr1 Ccdc162 Sh3tc1 Usp45 Olfr550 Pex12 Prrc2b Scarb2 Lonrf2 Il5ra Nfia Rpl5 Trmt12 Pcnx Ncl Bend7 Ptpro Lrp8 Stoml3 Hist2h2bb Edil3 Dnajc15 Zfp663 F2 Igfl3 Tpp1 Btg1 Jrkl Calcoco1 Cst8 Psma8 Gucy1b3 Gstm7 Galnt3 Abhd6 Herc4 Ppdpf Srp54b Phkg1 Wfdc1 Gm15093 Zswim5 Znrd1as Abt1 Cntnap5c Fibin Dct Clca6 Fam102b Vmn2r42 Pcdhb19 Asic5 Eml1 Gm15446 Aff2 Abi1 Mycbp2 Ccdc88a Themis2 Pilrb2 Odf3l1 Zcchc2 Gpatch2 Cdh10 Vwce 1700123O20Rik Ppp1r3a Kank4 Dnajc18 Hyal4 Pdk2 Sh2d1b2 Eef1e1 Nipbl Ugt8a H2-Q1 D19Bwg1357e Fyn Aste1 Neurl1b Prr5 AU041133 Smpx Hfe Wdr66 Gm11007 Dtymk Ppm1d Oprd1 Mmd Asb10 Pfkp Unc5d Serpina1c Slc6a12 Cflar Srgn Heg1 Bdnf Kdm4b Cmah Cenpc1 Aven Ephb4 Foxi3 2510039O18Rik Aqp7 Ncan Plekho1 Mepce Fbxo39 Kctd12 6430548M08Rik Bace1 Micu3 Kdm5a Tspyl5 2610305D13Rik Hace1 Tmco5b Ap5z1 9030624G23Rik Amot Ecm2 Golga3 Ddb2 Dsc2 Kif14 Slc2a12 Fgd4 Sertad2 Tfap2a Fbrs Loh12cr1 Tmem173 Jak1 Tmprss11b Hsf3 RSLCAN18 Stbd1 Nip7 Ddx6 Arfip2 Acer3 Slc30a4 Ammecr1 Hinfp Doc2b Htr7 Slc25a32 Ubtfl1 Kidins220 Fli1 Twist1 Tbp Rc3h1 2610021A01Rik Crp Mrpl19 Fam168a Chd9 Rbm4 Mocs2 Tmem2 Slc24a4 Lrrc66 Elovl7 Lclat1 Gpr141 1700016D06Rik Bambi Pcdhb17 Cnr2 Pigc Sprr2a2 Kbtbd8 Cebpzos 9430016H08Rik Pank3 Hist1h3g Ubn1 Kras Taco1 Zfp457 Rnmt Aebp2 Mgam Esco2 Loxl4 Pkn2 Gfm2 Mapre1 Tenm1 Fam134b Mrpl36 Manf Srd5a3 Gm6871 Nkx2-2 Rsc1a1 Hnf4g Hnrnpll Zfp872 Klhl20 Klf11 Rgs2 E030018B13Rik Pcdhb3 Aldh3b1 Rwdd4a Med13 Zdhhc24 Timm23 Uhmk1 Uox Zfp280b Dars Ppp1r26 Cst3 Gpr183 Bckdhb Serf1 Gja1 Lysmd2 Setmar Fer Ccdc129 Ppapdc3 Eef1a2 Uba3 Proser1 Xlr4a Lbr Cgref1 Rpp30 Chordc1 Yipf6 Ctr9 9230104L09Rik Cpne8 Apba1 Mon2 Stk17b Ahr Acy3 Dph2 Ero1l Slfn4 Dsg1b Spink3 Ly6f Ube3b Rnf11 Zcchc16 Gna12 Pik3cb Tmem41b Rbm26 Gnrh1 Gm10116 Amph Matn3 Fgf18 Mocs1 Nfu1 Dock7 Mfap1b Nek2 Atg4c Dkk3 Gpr45 Snx13 Ndst4 Slc22a22 Scn9a Clk4 Tmem9b 4930433I11Rik Olfr212 Gm3055 Gm15128 Slc2a8 Serpina3m Rel Gpr116 Atad2 Gm10521 Fam136b-ps Mesdc2 Unkl Zfp169 Map6d1 Mmp24 Acmsd Slc40a1 Pitpna Cngb1 Gm10375 Tarsl2 Rsbn1l Slc7a6 Gm7489 Zfp442 Zcchc11 E430025E21Rik Rhpn2 Alyref Ccdc130 Gm15080 Clcn4-2 Plaa Eif3f Pak1ip1 Phf12 Mrps35 Nap1l1 BC061237 Fras1 Ghsr Grpel1 Prrc2c Trim32 Igf2r Pyroxd2 Apon Cd47 Gfpt1 Bola2 Gzf1 Gm10030 Gpm6b Galnt14 Tsc22d3 Ckap4 Kdm6a Defb1 BC007180 Epha4 Rnd3 Gm3573 Slc15a4 Grin2d Zmynd19 Uvssa Zfp708 Opa1 Clcn6 Msh2 Pigk Prr24 Slc25a4 Pycard Kmt2a Ranbp3l Aff3 Bdh2 Alox15 Asap2 Vmn2r41 Fam47e Gucd1 Oprm1 Ptges2 Sypl Fam46c Irf2bp2 Sec22c Lin9 Styx Ednra Zfp781 Ubxn2a Mlec Ahcyl1 Spats2 Impa1 Hnrnph3 Flt3 Pxmp2 Rsad1 Appbp2 2700097O09Rik Arid1a Trim21 Adap2 Tpt1 Hecw1 Zdhhc3 Vmn1r56 Cyp2s1 Dennd5b Inpp5j Manea Prrx1 Wnt9b Kctd9 Ccnt2 Dnlz Gpr174 Cep19 Dnpep Abhd14b Snrpe Klk7 Kcnmb4 Map3k2 Gabrb2 Dsg1c Nrg3 Prdm15 Xpo1 Mkl2 Sco1 Atp6v1d B930041F14Rik E230019M04Rik 2310011J03Rik Zfp273 Tlr13 Gpr108 Mrps2 Klf10 Tor1aip2 Homez Rnpepl1 Heatr6 Olfr912 Mrpl35 Impg2 Zfp846 Ssbp2 AI747448 Cnih1 Sacm1l Xcr1 Hook1 Nbeal1 Mre11a Ube2k 1810043H04Rik Marcks Zfp2 Rasgrf2 Serpina5 Sdhd Cldn16 Vmn2r30 Pcdhb4 Fgf7 Scd4 Aqp2 0610009O20Rik Rchy1 Wrnip1 Actn2 Xlr4b Safb Ing3 Vegfb Cetn3 Hhla1 Hk2 Rasl10b 6330403A02Rik Rabep1 Rpl7l1 Fbxo6 Hells Chsy3 Akr1c12 Upp2 Rbbp4 Orc4 Mid2 Kpna6 Fbxo16 Gm21292 Rab10 Macc1 Ints3 Tlr3 Nos1ap Sec61b Shisa5 Cenpl Erlec1 Timmdc1 Extl3 Il7r Pten Mark4 Mlkl Eno1 Pirt Gskip Plag1 Asb3 Sass6 Slc35a5 Zc4h2 Lax1 Elovl5 Spg7 Dok2 Nhej1 Ggt5 Pex5 Arid5a Prr3 Bivm Gm7221 Fgfr1op2 Casc4 Ntn4 Ang Large Hsd17b7 Gm12216 Snx29 Snrnp200 Cops2 Cxcl15 Mgrn1 Aff1 Ube3a Zwint Ranbp2 Foxo4 Exoc3 Cnpy3 Cpox Isl1 Synpo2 Gm21685 Ate1 Nkain2 Hip1r Rnf7 St14 Frmd6 Gpr137c Rbm41 Glg1 Hfe2 Tgfbr1 Slc43a1 Rbm46 Gdap1 Crebl2 Commd6 Aldh6a1 R3hcc1l Trim33 Fam175b Sit1 Pigl Tmc1 G2e3 Mrpl47 Grip1 Wisp3 Gm14327 Colec12 Lrrc39 Yme1l1 Robo4 Aim2 Unc45b Mtmr7 Mbtps2 6330416G13Rik Zfp664 Dclre1a Zfp963 4930430A15Rik Krba1 9930021J03Rik Pnmal1 Atp1b4 Csrnp1 Smarcal1 Psmg4 Lurap1l Tmem52b Dhrs9 Ak4 Gm6034 Sdha Ppp3cc 4930522L14Rik Pcdhb1 9230110C19Rik Jmjd4 Vmn2r37 Arhgef38 Slc35f5 Lama3 Cep41 4932415D10Rik Dusp28 Il18bp Slc22a19 Pcdhb14 Nxpe4 Zfp709 Gpr150 Samd12 Rccd1 4930523C07Rik BC005537 Rab39 Gm15091 Trpc5 Ccdc104 Ankrd22 Myh10 Ddx11 Psd2 Cog1 Cdk17 Lrp2bp Pard6b Gm10704 H2-M10.2 2610008E11Rik Phex Jph1 Hadhb Tbx20 Tmem160 Gm15085 Msmo1 Pik3r6 Capza1 F2rl1 Zkscan17 Alad Tmem167 Rnf14 Tmem80 Scarf2 Sec22a Vps13b Sdc1 Trappc11 Ddx10 Zkscan8 E030037K01Rik Megf6 Noc3l Pou2af1 Abcd3 Plekha1 Dram1 Utp6 Prr12 Entpd4 Nsun6 Tbk1 Psg18 T Srpk1 Hsbp1 Hspa4l Sntb2 Esp15 Nrde2 Gopc Gm5616 Acss1 8030462N17Rik Ddx3y Magi3 1700018B24Rik Gm14322 Dnase1l3 Smc5 Ddost Slc30a2 Grm5 Emc3 Kcnk18 C78339 Tmem210 2310039H08Rik Hipk2 Anp32e Rpl21 Npy2r Paqr4 Pnpla8 Tmem182 Nhp2l1 Lamp5 Astn2 Mbnl2 Gm5444 Pfas Twf1 Cep72 Gm3676 Stc2 Ccnyl1 Snx16 Gpatch8 Ankrd50 Gm12185 Zfp191 A730061H03Rik Efhd2 Tbx5 Xylb Jmjd6 Iba57 Gm13011 Ier5l Nwd2 Ccdc148 Rpl28 Gm8773 Zfp85 Clvs1 Rap1b Ercc3 Hdgfrp3 Zfp37 Abhd17c Mesp1 Tyw1 Cnep1r1 Inpp5f Enpp6 Clec7a Foxi1 Htr1f Slc35a3 Mob4 Gm14296 Serpina7 Atm Uroc1 Adra1d Pacrg Tmem229a Folh1 Ddi2 Zfp30 Cd44 Stx12 A430033K04Rik 1700013H16Rik Fuca1 Ttll7 Sike1 Ugdh Ppef1 Noxred1 Mmrn2 Ndufa10 Ankrd33 Hipk1 Aasdhppt Nr1h4 Htr2a Luzp4 Olfr920 Plod3 Foxo3 Il17d Dhx40 Pold4 Dcaf10 Tmem237 Thsd4 Peg3 Rcan2 Runx3 Uap1l1 Rlim Mtnr1a Cdh19 Trim62 Fam188b Naa30 Itga2b Pex13 AU019823 Tpcn2 Tpte Spesp1 Crym Trappc12 Tspan13 Fabp12 Itpr2 Zfp964 Hsd17b6 Habp4 Mstn Clec5a Brwd1 Eif2ak2 BC048403 Wnt10a Txndc16 Ube2s Zranb2 0610037L13Rik Phb2 Lage3 Cep85l Heph Mme Ptgfr Rab38 Ipo9 Mdfic Bdkrb2 Letm1 Stc1 Lipt2 Ccdc58 Ikzf5 Sf3b3 Slc17a3 Catsperb Gm7293 Pmf1 Olfr482 Arhgap5 B3gnt2 Nkx3-1 Rnf215 Ppp2r3c Gyk Brcc3 Ythdf2 Slirp Gbp5 Ppp1r3f Nf1 Syt4 Guf1 Fsd1l 1110002L01Rik Fbxl17 Cox8b C1qtnf6 Wnt5a Mkln1 Lrrc38 Bri3bp Bag5 Dnmt3a Arid3a Rrp8 Tceanc2 Yae1d1 Uhrf1bp1l Sptssb Fbxo32 Slamf1 Dcp2 Fyco1 Lpar4 1700023E05Rik Srsf10 Asb18 Ubap2 Gm5084 Cav2 Rad54b Mrps5 Oas1h Stag3 2510002D24Rik Tlr8 Serpinb1c Serpina12 Ube2i |

Further information and requests for reagents may be directed to, and will be fulfilled by, the Lead Contact, Jun-An Chen (jachen@imb.sinica.edu)
