# Supplementary material for: MicroRNAs mediate precise control of spinal interneuron populations to exert delicate sensory-to-motor outputs
Source: eLife. 2021 Mar 31;10:e63768. doi: 10.7554/eLife.63768 (PMC8075582; doi:10.7554/eLife.63768)
Supplement: Transparent reporting form [file elife-63768-transrepform.docx]

***eLife’s* transparent reporting form**

We encourage authors to provide detailed information *within their submission* to facilitate the interpretation and replication of experiments. Authors can upload supporting documentation to indicate the use of appropriate reporting guidelines for health-related research (see [EQUATOR Network](http://www.equator-network.org/%20)), life science research (see the [BioSharing Information Resource](https://biosharing.org/)), or the [ARRIVE guidelines](http://www.plosbiology.org/article/info:doi/10.1371/journal.pbio.1000412) for reporting work involving animal research. Where applicable, authors should refer to any relevant reporting standards documents in this form.

If you have any questions, please consult our Journal Policies and/or contact us: [editorial@elifesciences.org](mailto:editorial@elifesciences.org).

**Sample-size estimation**

- You should state whether an appropriate sample size was computed when the study was being designed
- You should state the statistical method of sample size computation and any required assumptions
- If no explicit power analysis was used, you should describe how you decided what sample (replicate) size (number) to use

Please outline where this information can be found within the submission (e.g., sections or figure legends), or explain why this information doesn’t apply to your submission:

Usually, at least three independent biological experiments were harvested for the designed molecular/biochemistry experiments. For the behavioral assays that require more replicates, we applied at least six mice for all behavioral tests.

**Replicates**

- You should report how often each experiment was performed
- You should include a definition of biological versus technical replication
- The data obtained should be provided and sufficient information should be provided to indicate the number of independent biological and/or technical replicates
- If you encountered any outliers, you should describe how these were handled
- Criteria for exclusion/inclusion of data should be clearly stated
- High-throughput sequence data should be uploaded before submission, with a private link for reviewers provided (these are available from both GEO and ArrayExpress)

Please outline where this information can be found within the submission (e.g., sections or figure legends), or explain why this information doesn’t apply to your submission:

The replicate number and quantification method for each experiment were detailed and specified in the figure legends. The technical replicates were described in the “Materials and Methods”.

**Statistical reporting**

- Statistical analysis methods should be described and justified
- Raw data should be presented in figures whenever informative to do so (typically when N per group is less than 10)
- For each experiment, you should identify the statistical tests used, exact values of N, definitions of center, methods of multiple test correction, and dispersion and precision measures (e.g., mean, median, SD, SEM, confidence intervals; and, for the major substantive results, a measure of effect size (e.g., Pearson's r, Cohen's d)
- Report exact p-values wherever possible alongside the summary statistics and 95% confidence intervals. These should be reported for all key questions and not only when the p-value is less than 0.05.

Please outline where this information can be found within the submission (e.g., sections or figure legends), or explain why this information doesn’t apply to your submission:

For the statistical analysis, we described the method in the section of “Materials and Methods”. The statistical approach used for each figure was specified in the figure legends. All the statistical significances less than 0.05 were all denoted as one asterisk mark in the figures.

(For large datasets, or papers with a very large number of statistical tests, you may upload a single table file with tests, Ns, etc., with reference to sections in the manuscript.)

**Group allocation**

- Indicate how samples were allocated into experimental groups (in the case of clinical studies, please specify allocation to treatment method); if randomization was used, please also state if restricted randomization was applied
- Indicate if masking was used during group allocation, data collection and/or data analysis

Please outline where this information can be found within the submission (e.g., sections or figure legends), or explain why this information doesn’t apply to your submission:

Group allocation is this study is based on the collection of different tissues/time points, as well as the mouse genotype with the same age. The control groups used for the comparison in each experiment were described in the figure legends. Owing to the complexity of littermate controls from the breeding of TKO/DKO, we combined different littermate controls together for the quantification, which was furthered mentioned and discussed in the “Result”, “Discussion”, and “Materials and Methods”. Control groups are further listed in the Supplementary file 1f.

**Additional data files (“source data”)**

- We encourage you to upload relevant additional data files, such as numerical data that are represented as a graph in a figure, or as a summary table
- Where provided, these should be in the most useful format, and they can be uploaded as “Source data” files linked to a main figure or table
- Include model definition files including the full list of parameters used
- Include code used for data analysis (e.g., R, MatLab)
- Avoid stating that data files are “available upon request”

Please indicate the figures or tables for which source data files have been provided:

The source data from the available online database were provided in the “Materials and Methods”. The mature sequences of miR-34/449 were collected from online “miRbase” and denoted in Figure 1A. Predicted targets for miR-34/449 from “TargetScan” was computed in “DAVID” (GO analysis) and displayed in Figure 5A. Gene lists used for the further analyses are provided in the Supplementary file 1g. R Script developed for this paper is provided in the “Data availability” section.
